# Genome-wide association study on blood pressure traits in the Iranian population suggests *ZBED9* as a new locus for hypertension

**DOI:** 10.1038/s41598-021-90925-w

**Published:** 2021-06-03

**Authors:** Goodarz Kolifarhood, Siamak Sabour, Mahdi Akbarzadeh, Bahareh Sedaghati-khayat, Kamran Guity, Saeid Rasekhi Dehkordi, Mahmoud Amiri Roudbar, Farzad Hadaegh, Fereidoun Azizi, Maryam S. Daneshpour

**Affiliations:** 1 Department of Epidemiology, School of Public Health and Safety, Shahid Beheshti University of Medical Sciences, Tehran, Iran; 2 Cellular and Molecular Research Center, Research Institute for Endocrine Sciences, Shahid Beheshti University of Medical Sciences, Tehran, Iran; 3 Department of Animal Science, Safiabad-Dezful Agricultural and Natural Resources Research and Education Center, Agricultural Research, Education and Extension Organization (AREEO), Dezful, Iran; 4 Prevention of Metabolic Disorders Research Center, Research Institute for Endocrine Sciences, Shahid Beheshti University of Medical Sciences, Tehran, Iran; 5 Endocrine Research Center, Research Institute for Endocrine Sciences, Shahid Beheshti University of Medical Sciences, Tehran, Iran

**Keywords:** Chronic kidney disease, Cardiovascular diseases, Arrhythmias, Hypertension

## Abstract

High blood pressure is the heritable risk factor for cardiovascular and kidney diseases. Genome-wide association studies(GWAS) on blood pressure traits increase our understanding of its underlying genetic basis. However, a large proportion of GWAS was conducted in Europeans, and some roadblocks deprive other populations to benefit from their results. Iranians population with a high degree of genomic specificity has not been represented in international databases to date, so to fill the gap, we explored the effects of 652,919 genomic variants on Systolic Blood Pressure (SBP), Diastolic Blood Pressure (DBP), and Hypertension (HTN) in 7694 Iranian adults aged 18 and over from Tehran Cardiometabolic Genetic Study (TCGS). We identified consistent signals on *ZBED9* associated with HTN in the genome-wide borderline threshold after adjusting for different sets of environmental predictors. Moreover, strong signals on *ABHD17C* and suggestive signals on *FBN1* were detected for DBP and SBP, respectively, while these signals were not consistent in different GWA analysis. Our finding on *ZBED9* was confirmed for all BP traits by linkage analysis in an independent sample. We found significant associations with similar direction of effects and allele frequency of genetic variants on *ZBED9* with DBP (genome-wide threshold) and HTN (nominal threshold) in GWAS summary data of UK Biobank. Although there is no strong evidence to support the function of *ZBED9* in blood pressure regulation, it provides new insight into the pleiotropic effects of hypertension and other cardiovascular diseases.

## Introduction

High blood pressure as a heritable risk factor with direct and indirect effects on the incidence of cardiovascular diseases (CVD), stroke, and chronic renal failure is the leading cause of morbidity and mortality worldwide^1,2^. In the analysis of world data, one in three adults is expected to be affected by high blood pressure up to 2025, while macro or microvascular complications are not limited to extreme ranges of systolic and diastolic blood pressures (SBP and DBP)^3^. Elevated BP is categorized into primary and secondary hypertension based on disease etiology. While the latter is secondary due to other diseases, some medications, and related side effects, the former is known as essential hypertension and is prevalent in all populations^4,5^.

Genetic and environmental risk factors such as age, obesity, and lifestyle are attributed to the heterogeneity of essential hypertension risk in the general population^6,7^. Emerging new genotyping technologies has facilitated studies with Genome-Wide Association (GWA) design in non-communicable and infectious diseases to detect risky or preventive genetic variants after adjusting for environmental risk factors. Promising results from these hypothesis-free studies addressing different pathophysiologic pathways and gene targets for future pharmacologic interventions in complex diseases^8^.

The majority of the genomic findings on BP traits show pleiotropic associations. Until recently, these findings were predominantly from Europe-centric studies, have not been translated into understanding of underpinning molecular pathways, new drug development and personalization of HTN treatment in diverse populations with different genetic backgrounds^9,10^.

From the viewpoint of genomic architectures, different distribution of risk allele frequencies are contributed to the non-generality of findings between ethnicities^11^. A recent review addressed evidence concerning the origin of non-generality of GWAS findings on BP traits in different ethnic groups. Accordingly, a large proportion of GWAS was conducted in individuals with European ancestry, and it was introduced as a major cause of low reproducibility of findings in other populations^12^.

Iran has experienced the epidemiological transition from communicable to non-communicable diseases during the last four decades, and higher prevalence rates of pre-hypertension (47.3%) and hypertension (22.6%) have been reported in both adults and adolescents in recent years^13,14^. High blood pressure is main risk factor for CVD events in the Iranian population and its prevalence among CVD cases aged over 18 years is between 17.3% to 20%^15,16^. In comparing super populations in 1KG project including, Europeans, East Asians, South Asians, Africans and Hispanics, a large number of novel variants with a higher degree of genomic specificity were identified in the Iranian population^17^. Heritability estimations on BP traits provide evidence to support role of genetic variants are playing on BP regulation in this population(*h*^2^_SBP_: 25%, *h*^2^_DBP_: 26%)^18^. However, the Iranian population has not been represented in international genomic databases to date, so there is no evidence concerning the effect(s) of genetic variants in this population. To fill the gap, we conducted the first study using GWA design to investigate the replication or discovery of genetic variants on the Iranian population's BP traits.

## Material and methods

### Study subjects

The Tehran Cardiometabolic Genetic Study (TCGS) is a family-based genetic analysis of the Tehran Lipid and Glucose Study (TLGS) as the oldest Iranian cohort. In total, more than 20,000 participants are followed in 7 phases since 1999 and underwent a clinical examination on more than 230 metabolic-related traits in each phase of the study. The Medical Ethics Committee of Shahid Beheshti University of Medical Sciences approved this study and all methods were performed in accordance with the relevant guidelines and regulations. All participants gave written informed consent to participate in the original cohort and TCGS. In the case of younger participants, written formal consent was obtained from the parents or guardians. Principles for clinical investigations could be found in the original TCGS and TLGS papers^19,20^.

In the present study, 17,462 participants from the five phases of TCGS (1999–2014) were recruited to investigate hypertension. More details on DNA sample collection, genotype quality control process, phenotype measurements, covariate imputation, case definition, and selection criteria are explained in the supplementary information 1.

### Study design

Based on critical points of BP changes in the age trajectory, all study subjects aged 18 or above were included in the present study, and average values of SBP and DBP during follow up visits were considered in GWA analysis. For binary trait analysis, HTN incident cases and a random sample of healthy individuals with two or more follow-up records were included in the analysis (Supplementary file, Figure S1, S2). Age, sex, Body Mass Index (BMI), Waist Circumference (WC), insulin resistance and top 5 PCs were included in both GWA analysis on quantitative and binary traits after imputing their missing values, using the Expectation-Maximization method with Bootstrapping (EMB) approach by Amelia package in R^21^ (Supplementary file, Figure S3).

### Quality control of genotypes

To maximize power against the removal of individuals and markers, quality control (QC) of genotyping data was implemented on a per-individual basis before per-marker using PLINK version 1.9 and R^22,23^. Accordingly, a standard QC pipeline on 652,919 SNP, with an average mean distance of 4 kilobases, were performed in 7694 adults after excluding Individuals with missing phenotype, discordance of genetically inferred sex versus self-report, genotype calls ≤ 10%, and high heterozygosity (F_statistics_ ± 3 standard deviation). Moreover, related subjects with Identity By Decent (IBD) ≥ 18.5% were excluded from the study. In the genotype level, variants with minor allele frequency (MAF) < 1%, missing genotype calls > 5%, and Hardy Weinberg Equilibrium (HWE) *P* value < 1e−6 for quantitative traits, *P* value < 1e−10 in the HTN cases and *P* value < 1e−6 in controls were filtered out (Supplementary file, Figure S4).

### Statistical analysis

After checking collinearity for all covariates (r^2^ > 0.8), linear and logistic regression tests were performed. To explore any associations between BP traits and genetic variants, the covariates and top 5 Principal Components(PC) were adjusted and additive and overdominant inheritance models on autosomal chromosomes were checked for GWAS on the quantitative and binary traits in PLINK v1.9, respectively. A conventional genome-wide threshold of 5 × 10^–8^ considered for a significant *P* value. The genomic inflation factor was computed for each analysis, and observed versus expected *P* values were highlighted in the Q–Q plots to check for population stratification. Finally, four regression-based multivariate analyses evaluated the predictive accuracy of initial GWAS outputs for quantitative traits on discovery dataset and also an independent sample of TCGS (N = 2799)^24^. Accordingly, Polygenic Risk Score (PRS) was calculated after adjusting for the covariates and top 5 PCs, to evaluate proportion of variance, which is explained by genomic variants (R^2^). We used a standard pipeline to evaluate R^2^ through calculating PRS on our GWAS summary data. Moreover, discrimination of best fit PRS was assessed for each analysis according to sex. The PRS was computed in the following steps by PLINK and R:

i. Pruning: A window size of 200 variants, sliding across the genome with step size of 50 variants at a time were considered, and any SNPs with r^2^ > 0.25 were filter out,
ii. Clumping: SNPs within 250 k of the index SNP were considered for clumping. Then, all SNPs are correlated with each other were removed (r^2^ > 0.2) with *P* value ≤ 1 and only index SNP captured,
iii. Seven *P* value thresholds (0.001, 0.05, 0.1, 0.2, 0.3, 0.4, and 0.5) for inclusion of SNPs in the PRS was extracted,
iv. PRS generated in different ranges of *P* values for SBP and DBP,
v. To find "best-fit" score for the quantitative traits, PRS calculated in different ranges of *P* values thresholds,
vi. R^2^ as an index of trait variance explained by genomic variants calculated in different ranges of *P* values thresholds for two sets of covariates, (including age, WC and Ins. resistance and top 5 PCs, and also age, BMI and Ins. resistance and top 5 PCs),
vii. Calculated R^2^ was plotted by different ranges of *P* values using "ggplot2",
viii. The best-fit PRS was plotted against quantitative traits based on best result of R^2^ by sex using "ggplot2".

### Confirmation study

The confirmation study was conducted on 1618 participants in 210 selected TCGS families with an age range of 1 to 93 and familial aggregation of two or more affected (HTN) cases^25^. Similar to initial GWAS, the QC processes were applied in the confirmation study. After removing Mendelian errors and pruning out SNPs in linkage disequilibrium (LD) with a r^2^ > 0.2, the effects of significant independent SNPs in the initial GWAS were tested in the presence of the same covariate sets using two-level Haseman-Elston regression model by SAGE version 6.4^26^. This model-free linkage analysis is mathematically equivalent to the likelihood-based score test in variance component linkage analysis, which compute a random effect for genetic variants with *T* score test.

### Post GWAS

Four consecutive steps were followed to explain probable functions and pathophysiologic pathway(s) of discovered variants' effects on BP with *P* values less than 1 × 10^–4^. In the first step, chromosomal coordinates, genes, transcripts, and variants on protein sequence were annotated in Ensemble Variant Effect Predicator^27^. In the second step, GWAS catalog information was retrieved to identify the association of specified loci on BP traits^28^. Moreover, we sought to map known BP loci by assessing these loci's functional consequences in Ensemble^29^. Functional analysis was performed for those variants with consistent results and at least a *P* value less than 5 × 10^–7^ after adjusting for different covariate sets. In the case of a similar locus, ldlink browser by National Cancer Institute and LinDA (LINkage Disequilibrium-based Annotation) browser were checked for LD of detected and previously reported variant(s) in all populations using the website http://analysistools.nci.nih.gov/LDlink/ and http://linda.irgb.cnr.it/, respectively. In the third step, a list of detailed information on all loci was retrieved separately in Open Targets POST GWAS, including disease associations, protein interactions, pathways, similar targets based on diseases in common, RNA, and protein baseline expression by the anatomical system and organ^30^. In the final step, the overall association score was retrieved for each locus and other genes whose protein products interact with new loci through protein interaction networks^31,32^. Open Target Platform provides the score from 20 data sources, is ranged from 0 to 1, that the former implies no evidence and later corresponds to the most reliable evidence supporting evidence based on frequency, severity, and significance of association^33^.

## Results

### GWAS datasets

Table 1 describes the phenotypes and covariates characteristics in three datasets of discovery and confirmation studies. After applying DNA samples and genomic markers metrics, 4657 individuals with 616,263 SNPs on the quantitative traits and 4214 individuals with 616,308 SNPs on the HTN trait passed genotype QC. The discovery sample had a larger proportion of normotensive individuals, whereas the proportion of affected and unaffected cases was balanced in the confirmation study. Further, there was similar distributions of covariates in discovery and confirmation datasets, while high collinearity between BMI and WC was highlighted; hence, these effects were adjusted in three different sets of covariates including, (1) age, BMI, Ins. resistance and top 5 PCs, (2) age, WC, Ins. resistance and top 5 PCs, and (3) age, sex and top 5 PCs (Supplementary file, Figures S5, S6).

**Table 1:** Descriptive characteristics of phenotypes and covariates in GWAS and confirmation datasets.

Variable	Discovery		Confirmation
	SBP/DBP	HTN	SBP/DBP/HTN
	Adults (N = 4657)	Adults (N = 4214)	TCGS families (N = 210) Fam. Members (N = 1618)
Age (years)	40.8 ± 15.9	40.0 ± 13.5	35.6 ± 19.4
Female (%)	2523 (54%)	2143 (51%)	834 (52%)
SBP (mmHg)	116.29 ± 17.61	112.61 ± 12.29	117.61 ± 18.94
DBP (mmHg)	76.21 ± 9.72	74.70 ± 7.62	77.36 ± 10.49
BMI (kg/m^2^)	27.07 ± 4.51	27.01 ± 4.28	24.45 ± 6.90
WC (cm)	90.82 ± 11.51	90.60 ± 10.72	86.06 ± 15.77
TG/HDL	3.81 ± 3.11	3.87 ± 2.08	–
HTN Case (%)	NA	970 (23%)	855 (53%)

Mean ± standard deviation, *NA* not applicable, *Fam. Members* family members.

### GWAS result

**Figure 1:**
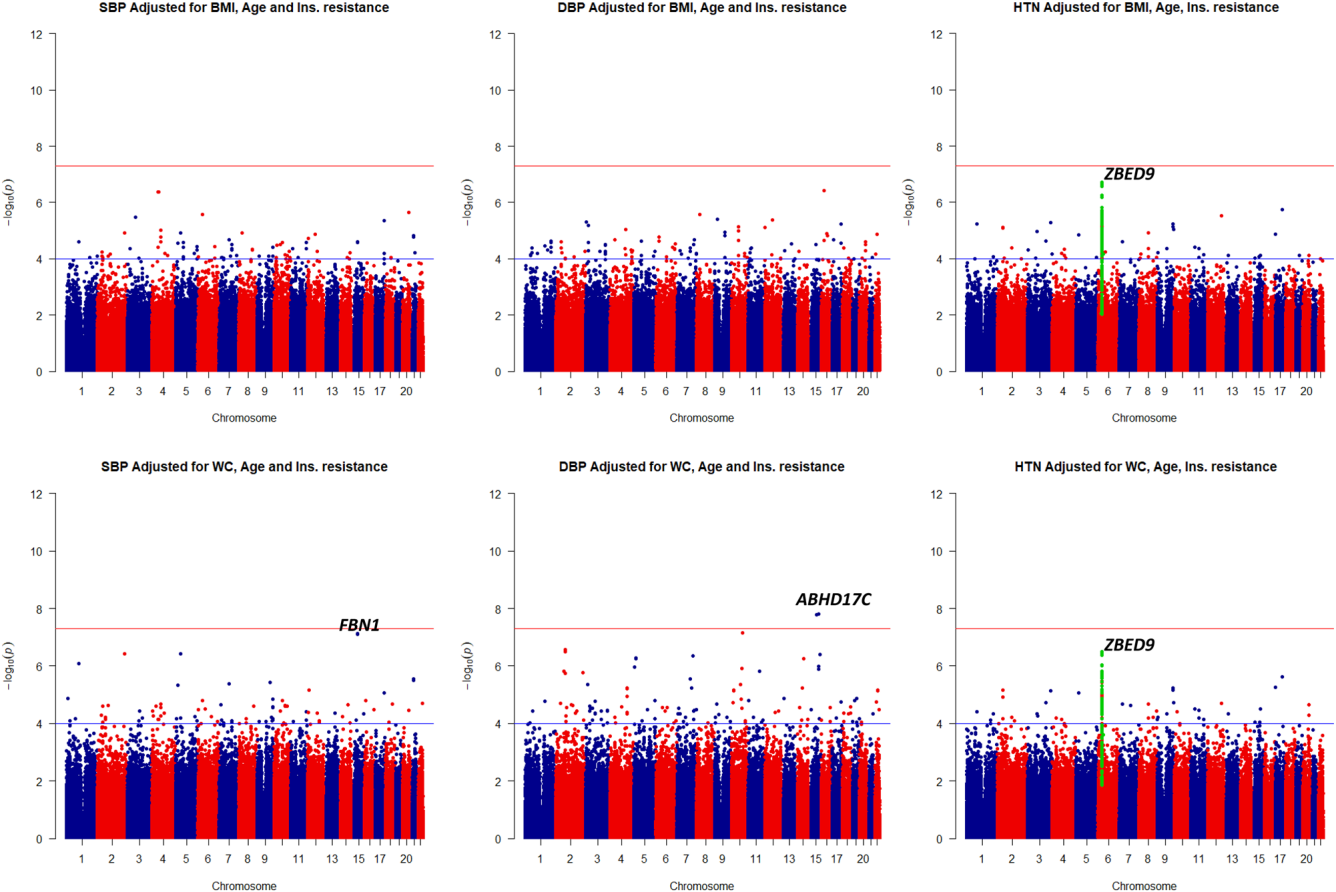
Manhattan plots of BP quantitative and binary traits, adjusted for two covariate sets of BMI and WC.

**Figure 2:**
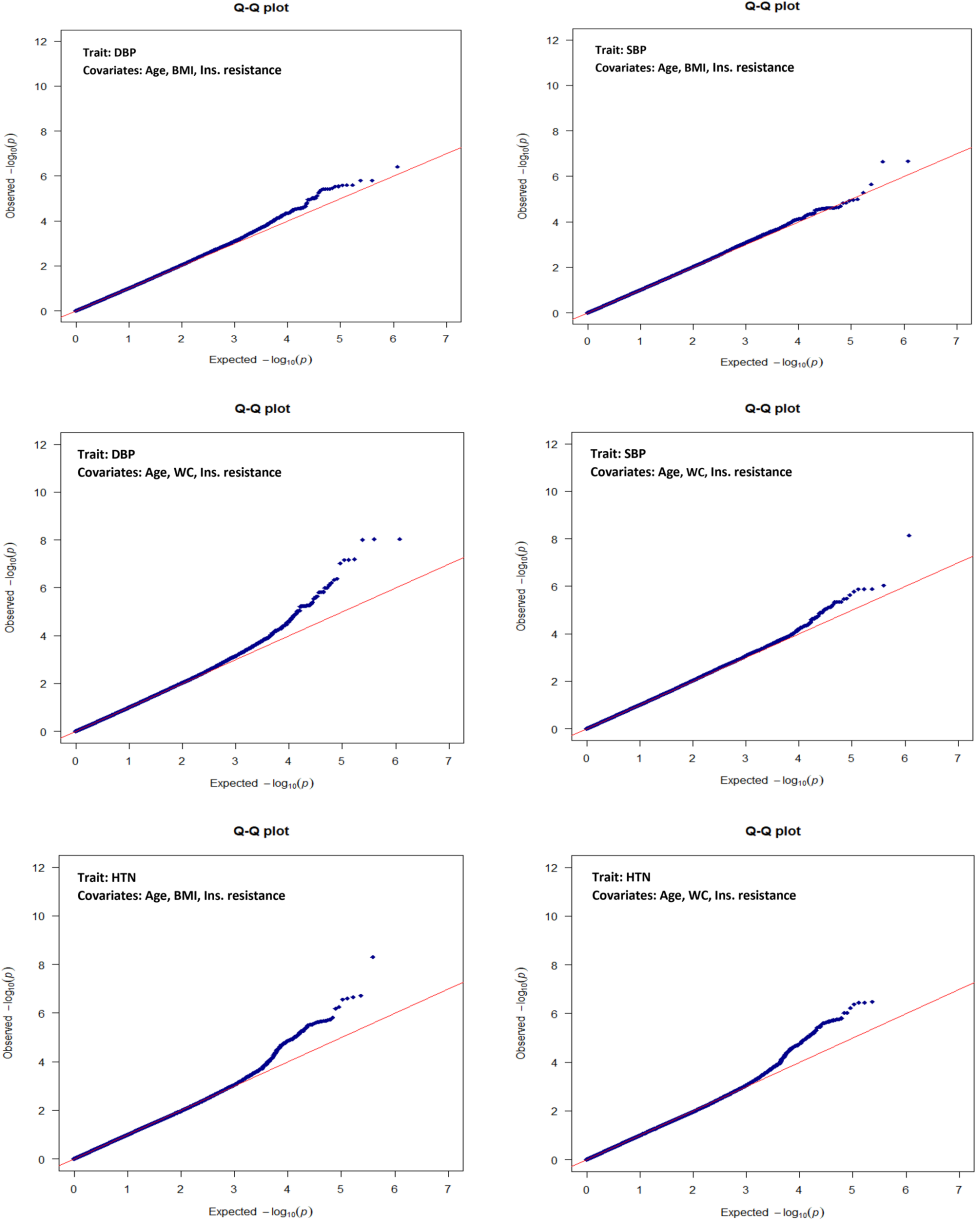
Q–Q plots of observed versus expected *P* values for BP quantitative and binary traits.

Table 2 describes GWAS findings in the discovery and confirmation studies. In the genome-wide analysis of quantitative traits, two signals on *ABHD17C* detected for DBP corresponding to the genome-wide significance threshold. Moreover, suggestive signals within 6p22.1 (*ZBED9*) and *FBN1* with borderline *P* values detected with the HTN and SBP traits, respectively (Fig. 1). The detected variants on *ZBED9 and ABHD17C* associated with HTN and DBP by two different sets of covariates, respectively. Moreover, *P* values for detected SNPs on *ZBED9* were slightly attenuated after adjusting for age, sex and top 5 PCs (Supplementary file, Table S1). The test statistic of genomic inflation was low for both analyses on quantitative and binary traits (λ: 1.001–1.01), and there was no population stratification (Fig. 2).

**Table 2:** GWA findings on BP traits in discovery and confirmation studies.

Trait	Locus nearby	Position	rs ID	Effect/other allele	EAF	Discovery		Confirmation			Reported locus (Ref)
						Adults (+ 18)		TCGS families			
						St. β (95% CI)	*P* value	*T* value	*P* value	Trait	
SBP	*FBN1*	15:48420560	rs2303505	T/G	0.02	0.37 (0.23–0.50)	7.45E−08^†^	0.44	0.24	**–**	Reported^34–37^
		15:48428455	rs363830	T/C	0.02	0.37 (0.24–0.51)	7.70 E−08^†^	NA	NA		
		15:48420679	rs363838	G/T	0.02	0.37 (0.23–0.50)	7.67 E−08^†^	NA	NA		
DBP	*ABHD17C*	15:80717774	rs1078107	C/T	0.01	0.10 (0.07–0.14)	1.55 E−08^†^*	0.09	0.17	**–**	Reported^34–36,38–40^
		15:80689205	rs16972291	C/T	0.01	0.10 (0.06–0.13)	1.58 E−08^†^*	NA	NA		
HTN	*ZBED9*	6:28574647	rs450630	A/G	0.43	0.64 (0.54–0.76)^#^	3.58 E−07^‡^	**4.03**	**0.02**	**SBP,DBP,HTN**	Reported^41^
		6:28611694	rs9501180	T/C	0.43	0.64 (0.54–0.76)^#^	4.13 E−07^‡^	NA	NA		
		6:28602172	rs380914	T/C	0.43	0.65 (0.55–0.77)^#^	9.41 E−07^‡^	NA	NA		
		6:28554918	rs6456825	G/A	0.43	0.64 (0.54–0.76)^#^	3.24 E−07^‡^	NA	NA		
		6:28682576	rs9885928	A/G	0.43	0.64 (0.54–0.76)^#^	3.76 E−07^‡^	NA	NA		

*EAF* effect allele frequency in TCGS, *St. β* standardized effect estimate, *CI* confidence interval.

*NA* not applicable due to removing variant in LD pruning process (r^2^ > 0.2**).**

^†^Reach the genome-wide borderline *P* value threshold after adjustment for WC.

*Reach genome-wide threshold after adjustment for age and sex.

^‡^Reach the genome-wide borderline *P* value threshold after adjustment for BMI/WC.

^**#**^Odds ratio.

In the confirmation analysis for detected SNPs in initial GWAS, the association of rs450630 on *ZBED9* was replicated for SBP, DBP, and HTN traits after LD pruning on other variants and adjusting for three covariate sets (*P* value < 0.05). The associations of other genetic variants on *ZBED9* with QRS amplitude and interval in Europeans and also *ABHD17C* and *FBN1* with BP traits or cardiovascular diseases in Europeans and East Asians were previously reported in GWAS literature. However, there is no correlation (LD) between detected and previously reported genetic variants in the same locus (Supplementary file, Figure S7).

In an inquiry of GWAS summary data on blood pressure related phenotypes in UK Biobank and also Japanese population, we found out similar associations (direction of effect and allele frequency) of detected variants on *ZBED9* with DBP (*P* value ≤ 5 × 10^–8^) and HTN (*P* value ≤ 1 × 10^–4^) in UK Biobank, while the associations were not significant (even in nominal threshold of 5 × 10^–2^) in Japanese population (Table 3). We checked out for LD of associated variants in 6p22.1 by LinDA. However, we could not find any reports in previous studies for association between correlated variants (r^2^ > 0.4) and BP traits in the region. Except for Africans, allele frequencies of detected variants on *ZBED9* are similar in Europeans, South Asians, Middle East(Qatari) and Iranian population, while the allele frequencies of detected variants on *FBN1* and *ABHD17C* is less than 1% in Europeans (Supplementary file, Table S2).

**Table 3:** Association of GWA findings on BP traits in UKbiobank and Japanese population.

Locus nearby	Position	rs ID	Effect/other allele	UKbiobank							Japanese pop				
				EAF	HTN		DBP		SBP		EAF	DBP		SBP	
					β (se)	*P* value	β (se)	*P* value	β (se)	*P* value		β (se)	*P* value	β (se)	*P* value
*FBN1*	15:48420560	rs2303505	T/G	0.008	7.2E−03 (5.5E−03)	1.9E−01	1.5E−02 (1.3E−02)	2.4E−01	2.3E−03 (1.2E−02)	8.4E−01	0.13	− 2.9E−03 (5.3E−03)	6.0E−01	− 6.3E−03 (5.6E−03)	2.6E−01
	15:48428455	rs363830	T/C	0.017	8.8E−03 (3.8E−03)	**2.1E−02**	2.3E−03 (9.2E−03)	7.9E−01	− 1.8E−04 (8.7E−03)	9.8E−01	0.13	3.0E−03 (5.6E−03)	5.9E−01	− 1.8E−04 (8.7E−03)	9.8E−01
	15:48420679	rs363838	G/T	0.02	7.0E−03 (5.5E−03)	2.0E−01	1.5E−02 (1.3E−02)	2.5E−01	2.6E−03 (1.2E−02)	8.3E−01	0.13	− 2.9E−03 (5.6E−03)	6.0E−01	− 6.3E−03 (5.6E−03)	2.6E−01
*ABHD17C*	15:80717774	rs1078107	C/T	0.003	− 1.3E−02 (8.4E−03)	1.0E−01	− 4.2E−02 (2.0E−02)	**3.2E−02**	− 2.6E−02 (1.9E−02)	1.6E−01	0.07	− 9.5E−02 (7.2E−03)	1.9E−01	− 4.7E−03 (7.2E−03)	5.1E−01
	15:80689205	rs16972291	C/T	0.003	− 1.2E−02 (8.7E−03)	1.6E−01	− 3.9E−02 (2.0E−02)	**5.6E−02**	− 2.5E−02 (1.9E−02)	2.0E−01	0.04	− 1.4E−02 (8.8E−03)	1.0E−01	3.3E−03 (8.8E−03)	7.0E−01
*ZBED9*	6:28574647	rs450630	A/G	0.46	− 3.6E−03 (1.0E−03)	**2.5E−04**	− 1.5E−02 (2.3E−03)	**2.4E−10**	6.2E−05 (2.2E−03)	9.7E−01	0.33	1.7E−03 (4.0E−03)	6.8E−01	5.0E−03 (4.0E−03)	2.1E−01
	6:28611694	rs9501180	T/C	0.47	3.9E−03 (1.0E−03)	**9.5E−05**	1.5E−02 (2.3E−03)	**2.3E−10**	− 1.3E−04 (2.2E−03)	9.5E−01	0.33	1.9E−03 (4.0E−03)	6.3E−01	5.0E−03 (4.0E−03)	2.1E−01
	6:28602172	rs380914	T/C	0.47	− 3.7E−03 (1.0E−03)	**1.6E−04**	− 1.4E−02 (2.3E−03)	**3.4E−10**	5.2E−04 (2.2E−03)	8.1E−01	0.33	1.9E−03 (4.0E−03)	6.4E−01	4.9E−03 (4.0E−03)	2.2E−01
	6:28554918	rs6456825	G/A	0.46	− 3.8E−03 (1.0E−03)	**1.1E−04**	− 1.5E−02 (2.3E−03)	**1.3E−10**	− 8. 9E−05 (2.2E−03)	9.6E−01	0.33	1.8E−03 (4.0E−03)	6.5E−01	5.1E−03 (4.0E−03)	2.0E−01
	6:28682576	rs9885928	A/G	0.49	− 3.6E−03 (1.0E−03)	**3.3E−04**	− 1.3E−02 (2.3E−03)	**2.3E−08**	− 1.2E−03 (2.2E−03)	5.8E−01	0.43	2.1E−03 (4.0E−03)	6.0E−01	5.7E−03 (4.0E−03)	1.6E−01

*EAF* effect allele frequency, *β* effect estimate, *se* standard error.

### Polygenic risk score estimation

**Figure 3:**
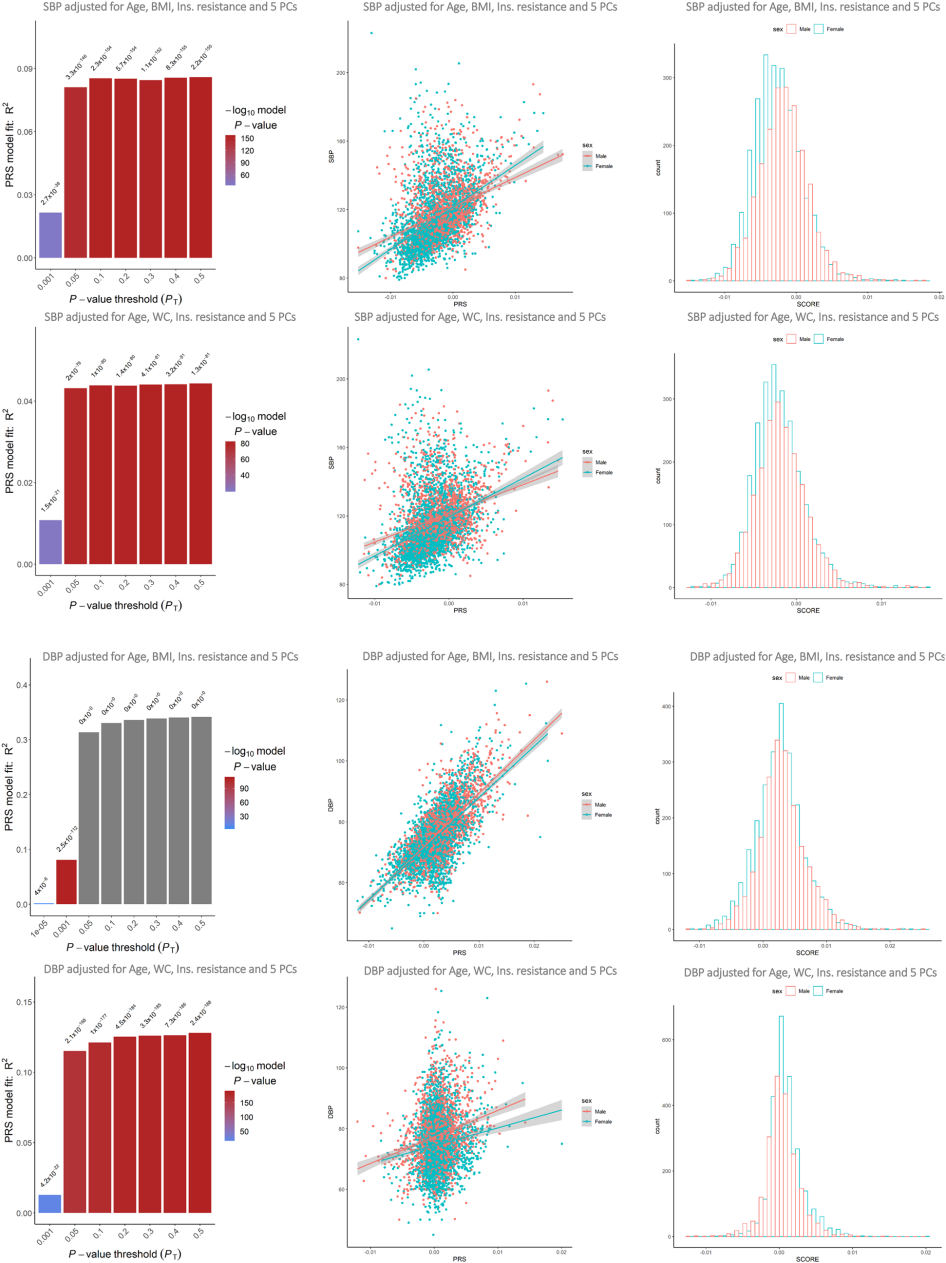
Predictive accuracy corresponding to a range of *P* value thresholds in regression models and the scatter plot of best fit PRS by sex in the discovery dataset.

**Figure 4:**
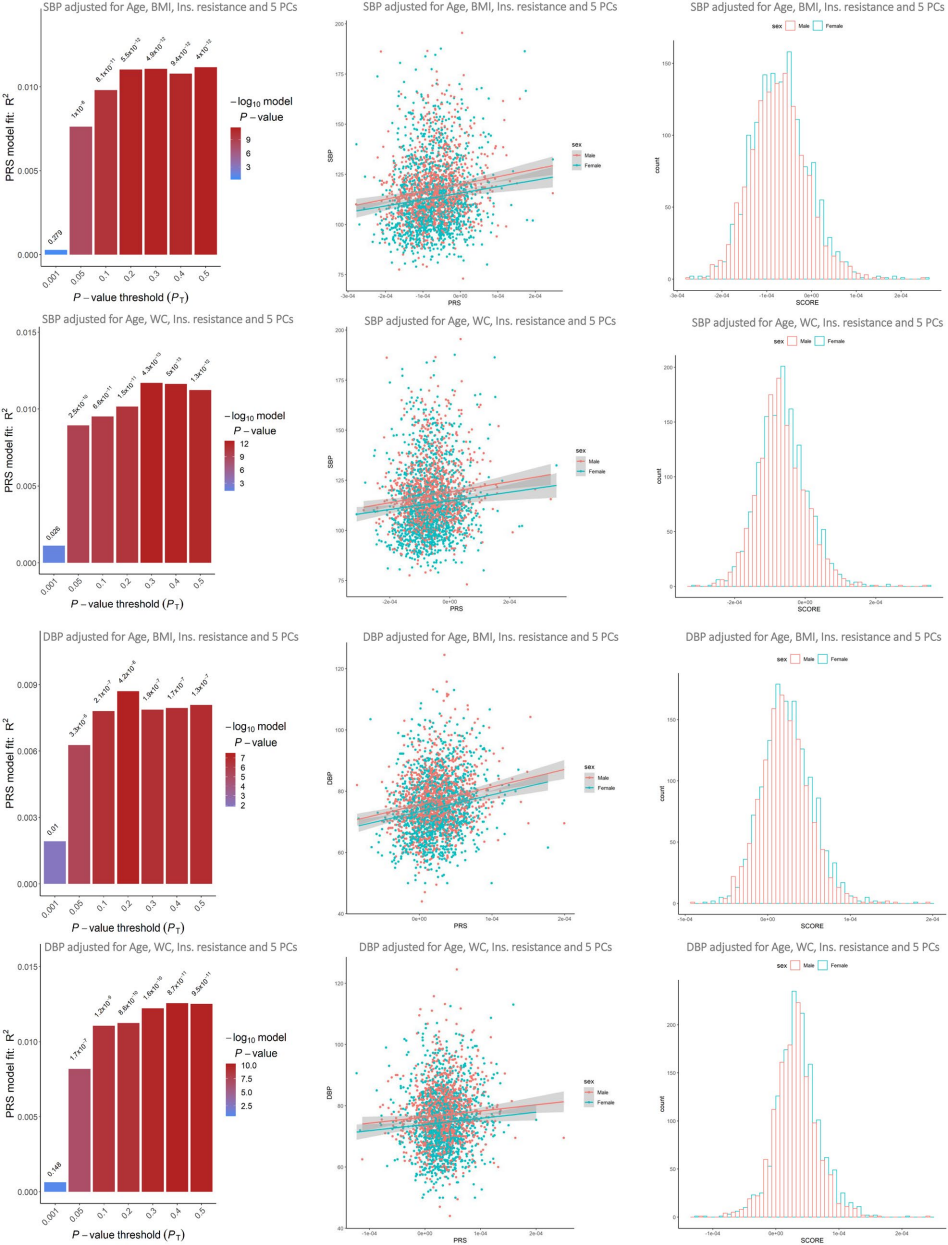
Predictive accuracy corresponding to a range of *P* value thresholds in regression models and the scatter plot of best fit PRS by sex in the independent sample of TCGS.

Multivariate analysis of PRS on initial GWAS outputs in the discovery dataset showed evidence of correlations between calculated PRS with SBP and DBP in three out of four models. The genomic variants account for 4.5–12.7% of the total variance of quantitative traits. Moreover, a similar distribution of PRS-trait correlation was seen by sex for each statistical model in discrimination analysis (Fig. 3). However, the calculated R^2^ in the independent sample of TCGS was much lower compared to discovery dataset (≤ 1%) and there was low correlation between the PRS and BP traits by sex (Fig. 4).

### Variant effects on protein-coding sequence

**Figure 5:**
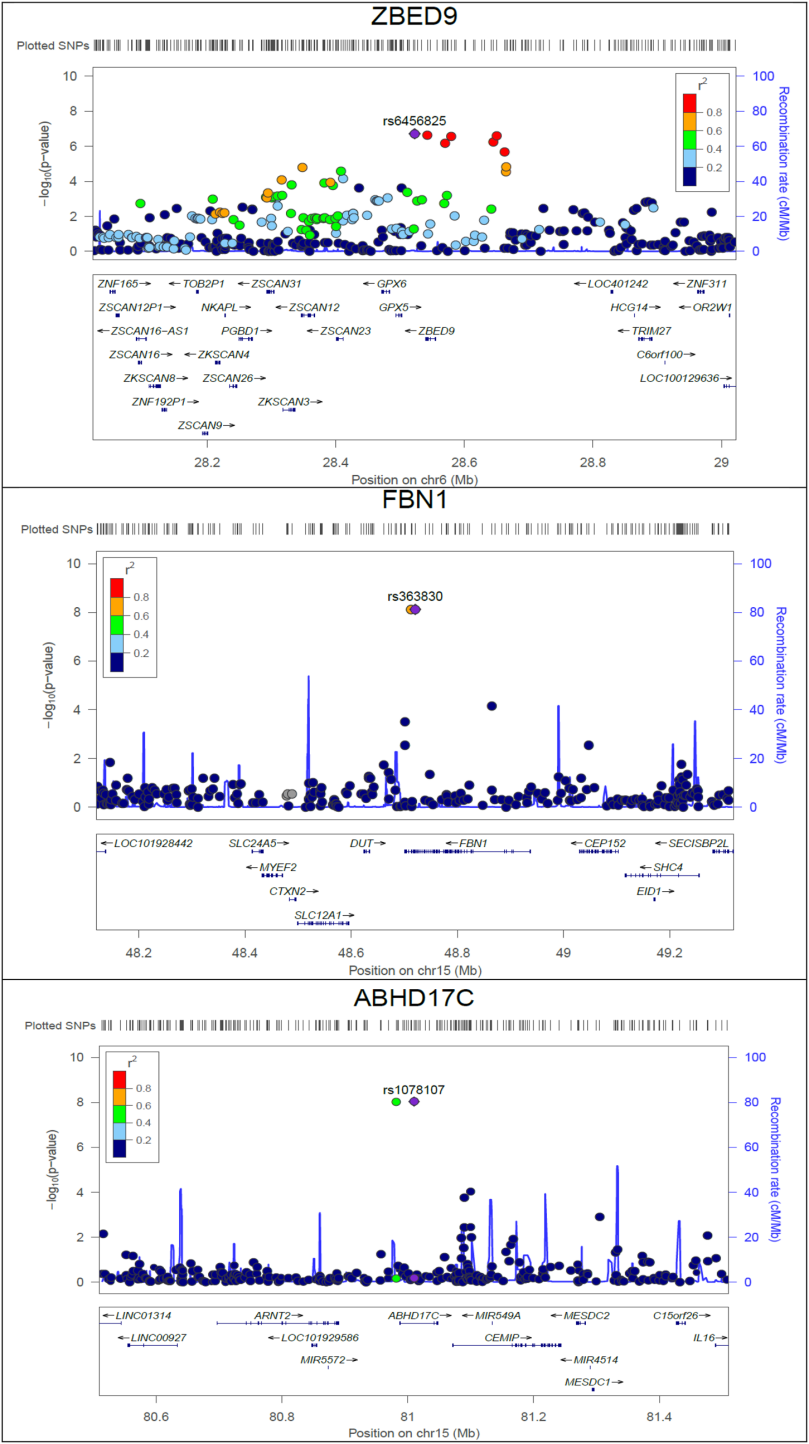
Locus zoom plot of GWAS findings in detected loci.

In the locus-based regulatory annotation, detected signals on *ABHD17C* and *ZBED9* were annotated as an intronic variant. All detected SNPs on the *FBN1* gene were in relatively high LD, while rs363830 and rs363838 were missense and splice variants. Also, two variants on *ABHD17C* were in moderate LD (r^2^: 0.4–0.6)*.* In further investigation via sentinel variants, we found rs853684 on *ZSCAN31* as a missense consequence in moderate LD (r^2^ > 0.60) and five missense variants in perfect LD (r^2^ = 1.0) with detected variants on *ZBED9* (Fig. 5).

### Prioritization of targets by scoring target-disease association

**Figure 6:**
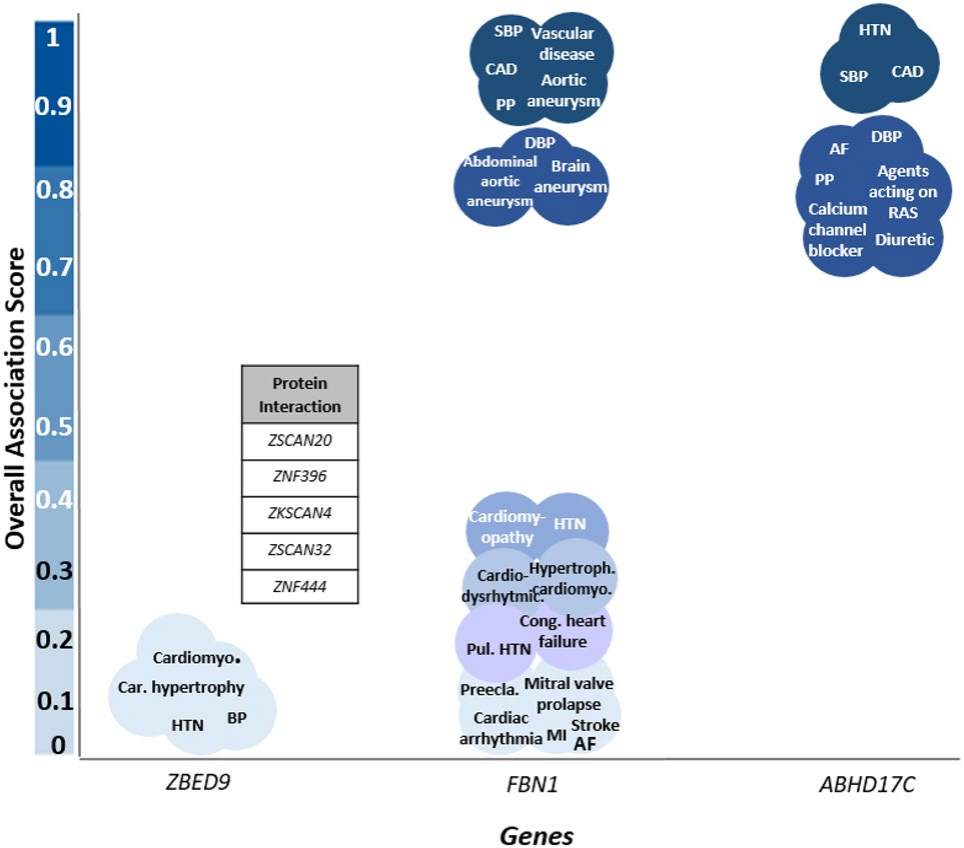
Prioritize targets by evidence of gene-disease overall association score in known loci and protein interaction networks of *ZBED9*.

The cumulative evidence for association with BP traits for three detected loci was retrieved in the Open Targets POST GWAS. The overall association scores were summarized in Fig. 6, with varying blue shades: the darker the blue, the stronger the association. Two previously reported loci of *FBN1* and *ABHD17C* to show evidence for a strong association with BP and cardiovascular disease traits, so they were prioritized as biological targets (overall association score = 1). Further, the protein interaction network suggests gene subnetworks for *ZBED9* with relatively low association with BP and cardiovascular diseases (overall association score ≤ 0.40), respectively.

## Discussion

To our knowledge, this is the first GWAS to examine genetic associations with BP traits in the Iranian population. This study identified suggestive and strong signals on *ZBED9*, *FBN1,* and *ABHD17C*, associated with HTN, SBP, and DBP, respectively. We found significant associations with similar direction of effect and allele frequency of detected variants on *ZBED9* with DBP (genome-wide threshold) and HTN (nominal threshold) in UK Biobank. However, There is no LD between detected and previously reported genetic variants on three loci in Europeans and other populations including, South Asian, Hispanic or Latin American, East Asian, African American, and Afro-Caribbean populations. The detected signals were not consistent after adjusting for two different sets of covariates on *FBN1* and *ABHD17C*. On the other hand, the association of genetic variants on *ZBED9* and BP traits has not been previously reported in other populations, while their effects were consistent after controlling for environmental factors by two different GWA analysis. Further, the confirmed association of genetic variants on *ZBED9* in discovery study with SBP, DBP, and HTN in an independent sample of TCGS families substantially increase our knowledge of its effects on HTN in the Iranian population.

In an effort to explain the function of new loci by RNA and protein baseline expression, the rate of expression for all detected loci was in the range of low to moderate in the circulatory system, including blood, endothelial cells of the umbilical vein, aorta, coronary artery, left heart ventricle, tibial artery, atrium auricular region, and heart muscle, provided by Human Protein Atlas and Expression Atlas^30^.

There is no evidence to support the functional effects of *ZBED9* on BP regulation in the literature. However, GeneHancer-gene association results indicate some known BP loci, including *EBF1*, *NR2F2*, *SOX5*, and *PRDM6*, are likely acting as transcription factor binding sites or gene targets for *ZBED9*^34–37,42–46^. In this way, significant association with QRS interval and amplitude as a surrogate of myocardial mass in European population^41^ and replication with SBP, DBP and also HTN using different criteria based on reference adjusted curves in the Iranian children and adolescents^47^, who was included in TCGS confirmation study is a signal for further investigations on the functional role of *ZBED9* in BP regulation pathway in other populations.

High blood pressure is directly associated with vascular mortality due to stroke, cardiomyopathy, aneurysm, cardiac hypertrophy, aorta stenosis, sudden cardiac arrest, and myocardial infarction^48^. Accordingly, gene-set-based analysis by Open Target Post GWAS highlighted the probable pleiotropic effects of detected loci, which are likely acting as a common genetic etiology for BP and cardiovascular diseases. Accordingly, the lethal cardiovascular diseases may affect allele frequency of effective variants with advancing age in our study due to survival reduction, so overlook the effect of functional variants in older groups as a competing risk^49,50^.

We acknowledge that some limitations are evident in our study. First, there is evidence for significant differences in the magnitude of genetic variants effects on high blood pressure between men and women^51^. However, there was inadequate statistical power to conduct GWAS by sex after removing related individuals in our study. Moreover, the calculated PRS for the quantitative traits showed similar patterns by sex. Second, despite the high predictive accuracy of PRS by genomic variants and confirm significant SNPs on *ZBED9* with family-based regression analysis, it was a case for overfitting due to selecting individuals with similar genetic background from the TCGS cohort. Accordingly, our findings validated in an independent sample of the Iranian population. However, our results still needs to be cross-validated in a different sample of the Iranian population as well^52^. Third, using a family-based design for GWAS has the advantages of complete robustness against genetic heterogeneity. However, family-based designs are likely biased due to population substructures, and association tests yield at the price of inflated type I error and reduce statistical power^53,54^. In this way, we included independent samples from a family bases cohort.

Additionally, there was no association between autozygosity and BP quantitative traits in an international meta-analysis, including TCGS families with a relatively high inbreeding rate^55^. Finally, we found signals which were not in LD with previously reported genetic variants on BP traits. Further investigation is necessary to fine mapping of these regions when imputed variants on the Iranian genome become available^56^.

## Conclusion

We identified three loci, of which two loci were previously reported in individuals with European and Non-European ancestries. The association of genetic variants on *ZBED9* and BP traits has not been previously reported in other populations. Their effects were consistent after controlling environmental factors by two different GWA analyses and confirmed in a family-based linkage study. Although there is no strong evidence to support the function of *ZBED9* in blood pressure regulation by Open Target Post GWAS, its association with QRS interval and QRS amplitude as surrogates of myocardial mass may provide new insight into pleiotropic effects of hypertension and other cardiovascular diseases.

## Supplementary Information

Supplementary Information 1.

Supplementary Information 2.

Supplementary Information 3.

## Data Availability

Summary-level data for BP traits in UK Biobank and Japanese population are publicly available in (http://www.nealelab.is/uk-biobank/) and (http://jenger.riken.jp/en/result), respectively.
