# Supplementary material for: Genome-wide association study on blood pressure traits in the Iranian population suggests ZBED9 as a new locus for hypertension
Source: Sci Rep. 2021 Jun 3;11:11699. doi: 10.1038/s41598-021-90925-w (PMC8175429; doi:10.1038/s41598-021-90925-w)

**Supplementary**

A genome-wide association study on blood pressure traits in the Iranian population suggests *ZBED9* as a new locus for hypertension

**Authors:**

**Goodarz Kolifarhood^1,2^, Siamak Sabour^3^, Mahdi Akbarzadeh^4^, Bahareh Sedaghati-khayat^5^, Kamran Guity^6^, Saeid Rasekhi^7^, Mahmoud Amiri Roudbar^8^, Farzad Hadaegh^9^, Fereidoun Azizi^10^, Maryam S Daneshpour^11^***

1. Goodarz Kolifarhood, MSc, Ph.D., Department of Epidemiology, School of Public Health and Safety, Shahid Beheshti University of Medical Sciences, Tehran, Iran. Email: [gfarhood@gmail.com](mailto:gfarhood@gmail.com)
2. Cellular and Molecular Research Center, Research Institute for Endocrine Sciences, Shahid Beheshti University of Medical Sciences, Tehran, Iran.
3. Siamak Sabour, MD, Ph.D., Department of Epidemiology, School of Public Health and Safety, Shahid Beheshti University of Medical Sciences, Tehran, Iran. Email: [s.sabour@sbmu.ac.ir](mailto:s.sabour@sbmu.ac.ir)
4. Mahdi Akbarzadeh, MSc, Ph.D., Cellular, and Molecular Research Center, Research Institute for Endocrine Sciences, Shahid Beheshti University of Medical Sciences, Tehran, Iran. Email: [akbarzadeh.ms@gmail.com](mailto:akbarzadeh.ms@gmail.com)
5. Bahareh Sedaghati-khayat, MSc, Cellular and Molecular Research Center, Research Institute for Endocrine Sciences, Shahid Beheshti University of Medical Sciences, Tehran, Iran. Email: [b.s.khayat@gmail.com](mailto:b.s.khayat@gmail.com)
6. Kamran Guity MSc, Cellular and Molecular Research Center, Research Institute for Endocrine Sciences, Shahid Beheshti University of Medical Sciences, Tehran, Iran. Email: [kamran.guity@gmail.com](mailto:kamran.guity@gmail.com)
7. Saeid Rasekhi, MSc, Cellular and Molecular Research Center, Research Institute for Endocrine Sciences, Shahid Beheshti University of Medical Sciences, Tehran, Iran. Email: [dehkordi.s.r@gmail.com](mailto:dehkordi.s.r@gmail.com)
8. Mahmoud Amiri Roudbar, MSc, Ph.D., Department of Animal Science, Safiabad-Dezful Agricultural and Natural Resources Research and Education Center, Agricultural Research, Education & Extension Organization (AREEO), Dezful, Iran. Email: [mahmood.amiri225@gmail.com](mailto:mahmood.amiri225@gmail.com).
9. Farzad Hadaegh, MD, Prevention of Metabolic Disorders Research Center, Research Institute for Endocrine Sciences, Shahid Beheshti University of Medical Sciences, Tehran, Iran. Email: [Farzadhadaegh@gmail.com](mailto:Farzadhadaegh@gmail.com)
10. Fereidoun Azizi, MD, Ph.D., Endocrine Research Center, Research Institute for Endocrine Sciences, Shahid Beheshti University of Medical Sciences, Tehran, Iran. Email: [azizi@endocrine.ac.ir](mailto:azizi@endocrine.ac.ir)
11. Maryam S Daneshpour, MSc, Ph.D., Cellular and Molecular Research Center, Research Institute for Endocrine Sciences, Shahid Beheshti University of Medical Sciences, Tehran, Iran. Email: [daneshpour@sbmu.ac.ir](mailto:daneshpour@sbmu.ac.ir)

*** Correspondence Author:**

Maryam S Daneshpour, Cellular, and Molecular Research Center, Research Institute for Endocrine Sciences, Shahid Beheshti University of Medical Sciences, Tehran, Iran; POBox: 19195-4763, Tel: +982122432500, Fax: +982122416264, Email: [daneshpour@sbmu.ac.ir](mailto:daneshpour@sbmu.ac.ir)

[Phenotype description](#_Toc54543718) 3

[Anthropometric](#_Toc54543719) 3

[Laboratory measurements](#_Toc54543720) 3

[Genotype evaluation](#_Toc54543721) 4

[DNA extraction](#_Toc54543722) 4

[Genotyping](#_Toc54543723) 4

[Relationship and drawing family tree](#_Toc54543724) 5

[Phenotype Calculations](#_Toc54543725) 5

[Body mass index (BMI)](#_Toc54543726) 5

[Tri-Ponderal Mass Index (TMI)](#_Toc54543727) 5

[Insulin resistance (Ins. resistance)](#_Toc54543728) 5

[The antihypertensive medication effect](#_Toc54543729) 5

[Data preparation strategy](#_Toc54543730) 6

[Quantitative traits](#_Toc54543731) 6

[Binary trait](#_Toc54543732) 6

[Covariates](#_Toc54543733) 6

[Data analysis](#_Toc54543734) 7

[Collinearity checking](#_Toc54543735) 7

[Population Stratification](#_Toc54543736) 7

[Polygenic Risk Score(PRS)](#_Toc54543737) 7

[Supplementary Figures](#_Toc54543738) 8

[Supp.1. Study flow diagram for phenotype and covariate data preparation.](#_Toc54543739) 8

[Supp.2. Distribution of SBP and DBP in five study phases by age.](#_Toc54543740) 9

[Supp.3. Sensitivity analysis of imputation values in five study phases (A-E)](#_Toc54543741) 10

[Supp.4. QC pipeline for quantitative and binary traits 1](#_Toc54543742)5

[Supp.5. Trait-covariate correlation. 1](#_Toc54543744)6

[Supp.6. Covariates correlation. 1](#_Toc54543744)7

[Supp.7. Previously reported associations between BP traits or cardiovascular diseases and… .](#_Toc54543749) 18

Phenotype description

Anthropometric

At the time of anthropometric measurements, the participants removed their shoes and wore light clothes. Waist circumference is measured at the umbilicus level, and hip circumference is measured over light clothing at the hip's widest girth. A qualified physician, after 15 minutes of participant arriving, measure blood pressure two times using a standard mercury sphygmomanometer calibrated by the Iranian Institute of Standards and Industrial
Researches. The participants remain seated for 15 minutes. A pediatric, regular adult or large cuff is used based on the participant's arm's circumference. The cuff is placed on the right arm, which is at the heart level, and inflated in as high a rate as possible increments until the cuff pressure is 30 mmHg above the level at which the radial pulse disappeared. There is at least a 30-second interval between those two separate measurements, and the mean of two measurements is recorded as the participant's blood pressure. The Systolic Blood Pressure (SBP) is defined as the first sound's appearance [Korotkoff phase 1]. The Diastolic Blood Pressure (DBP) is defined as the disappearance of the sound [Korotkoff phase 5] while deflating the cuff at a 2–3 mm per second decrement rate.

Laboratory measurements

After 12–14 hours of overnight fasting, a venous blood sample was drawn and centrifuged within 30–45 minutes of collection. All blood sampling was done between 7.00 and 9.00 a.m., and all measurements were completed on the day of sampling.

Fasting plasma glucose (FPG) was measured by the enzymatic colorimetric glucose oxidase method; inter-and intra-assay coefficients of variation (CV) at baseline and follow-up phases were both less than 2.3%. A Selectra 2 autoanalyzer (Vital Scientific, Spankeren, Netherlands) was used in the TLGS research laboratory on the day of blood collection to analyze samples for total serum cholesterol (TC) and TCGS. Enzymatic colorimetric tests were used to assay TC with cholesterol esterase and cholesterol oxidase; for TCGS, glycerol phosphate oxidase was used. HDL-C was measured after precipitation of the Lipoprotein-B-containing lipoproteins with phosphotungstic acid. Non-HDL-C was calculated by subtracting HDL-C from TC; TC/HDL-C and TG/HDL-C were calculated by dividing TC and TG by HDL-C, respectively. Both inter and intra-assay coefficients of variation were less than 1.9, 2.1, and 3% for TC, TG, and HDL-C, respectively, in all baseline and follow-up assays of lipid profile. Non-high density lipoprotein cholesterol (Non-HDLC) was calculated by subtracting HDL-C from TC. Low-density lipoprotein cholesterol (LDL-C) was calculated according to the modified Friedewald formula.

Genotype evaluation

DNA extraction

In the TLGS sample collection centre, a blood sample was drawn between 7:00 and 9:00 a.m. into vacationer tubes from all study participants after 12–14 hours overnight fast. Two blood samples were taken in a sitting position according to standard protocol. The blood collected in EDTA containing a test tube was used to immediately obtain DNA samples and immediately send it to the genomic laboratory. All the TLGS samples were recoded as a genomic sample connected to the TLGS code in a database. DNA was extracted from buffy-coat samples from each participant by the Proteinase K, salting out the standard method [15]. The quality and quantity of extracted DNA were evaluated. Thermo Scientific NanoDrop™ 1000 Spectrophotometer qualified samples were aliquot in 1.5 ml tubes and stored in -80°C ultra-freezers for future studies.

Genotyping

Parts of DNA samples were genotyped with HumanOmniExpress-24-v1-0 bead chips (containing 649,932 SNP loci with an average mean distance of 4 kb) at the deCODE genetics company (Iceland) according to the manufacturer's specifications (Illumina Inc., San Diego, CA, USA).

Relationship and drawing family tree

On the day of the examination, the TLGS participants were initially interviewed for their familial relationship. Genealogy data was drawn in Genepro (V 2.0.1.6) and checked by Family-Based Association Tests (FBAT-Toolkit V 1.7.3) [16]. Family data, pedigree information, phenotype, and genotype data were stored, manipulated, and error-checked using the genetic data management system (Progeny Clinical Version 7) from Progeny Software (Progeny Software LLC, Delray Beach, FL, www.progenygenetics.com). The father's name was checked for all individuals. If they have the same father name, their genetic relationship was checked with their genotype information. If their genetic relationship was confirmed with genotype information, their family tree joined together to make a mega family.

Phenotype Calculations

Body mass index (BMI)

The ratio of body weight to height squared (kg/m^2^) in adults (ages≥18).

Tri-Ponderal Mass Index in children and adolescents (for family-based confirmatory study)

The ratio of body weight to height cubed (kg/m^3^) in children and adolescents (ages ≤17).

Insulin resistance (Ins. resistance)

We are dividing the triglyceride concentration (TG) by high-density lipoprotein (HDL) levels as a surrogate marker associated with cardiometabolic risk.

The antihypertensive medication effect

We considered adding constant values of 15 and 10 mmHg to the SBP and DBP, respectively, for individuals with self-reported taking beta-blockers, calcium blockers, and diuretics.

Data preparation strategy for GWAS and Confirmation study

Quantitative traits

We checked the milestone of BP in a lifetime in five follow-up visits. Accordingly, we found two critical cut points in the age trajectory explained remarkable changes in both SBP and DBP. Therefore, we calculated the mean of SBP and DBP values during follow-up periods in three separate age groups of children (1-9yrs.), adolescents (10-17yrs.), and adults (>17 yrs.). The averaged values for children and adolescents were considered in a confirmatory study on TCGS families.

Binary trait

Due to substantial differences in BP traits distributions by age, sex, and height in European children and adolescents, the reference adjusted curves in the Iranian population rather US-4th-Report and German BP Percentiles (KiGGS) standards were considered for the confirmatory study. Accordingly, all children and adolescents (1-17 yrs.) with SBP and DBP percentiles ≥ 95th by sex, age, and height were classified as a hypertensive case^39^. Further, the following criteria were considered to define hypertension (HTN) in adults for GWA and confirmatory studies:

a) SBP ≥140 mmHg

b) DBP ≥90 mmHg

c) Taking antihypertensive medications.

Covariates

Risk factors of BP traits (SBP, DBP, and HTN) were chosen based on previous GWAS literature, and the following predictors were included: age, BMI, WC, and Insulin Resistance. Missing values of height were imputed based on age, sex, and parental and maternal height values. The remaining covariates' missing values were imputed simultaneously using age, sex, total cholesterol, fasting blood sugar, and Low-Density Lipoprotein (LDL) at each follow-up. Accordingly, the covariates were imputed multiple times using the Expectation-Maximization method with Bootstrapping (EMB) approach using Amelia package in R. Missing rate of covariate's values ranged from 0.2% to 7.7% over five follow up, with the highest and lowest missing values for WC and height in the second and fourth follow up.

In the sensitivity analysis on covariate imputation, except for height in the third and fourth follow-up and the lower bound of WC in all follow-up periods, the distribution of imputed values on remained covariates was overlayed on observed values. Small confidence intervals, well-behaved likelihood, and converge to the same point of starting values highlighted overall accurate imputation of the covariates' missing values.

Data analysis

Collinearity checking

All covariates were checked for collinearity by correlation plots using the "ggplot2" package in R. High collinearity between the anthropometric predictors were highlighted in adults.

Population Stratification

Observed versus expected *P* values of regression analysis were depicted for checking population stratification by Q-Q plots using the "*qqman*" package in R.

Polygenic Risk Score (PRS)

We computed PRS as a single value estimate of each participant's genetic liability to the quantitative traits. The PRS was calculated as the sum of their genome-wide genotypes, weighted by corresponding genotype effect size estimates derived from our GWAS summary statistic data.

Supplementary Figures

Supp.1. Study flow diagram for phenotype and covariate data preparation.


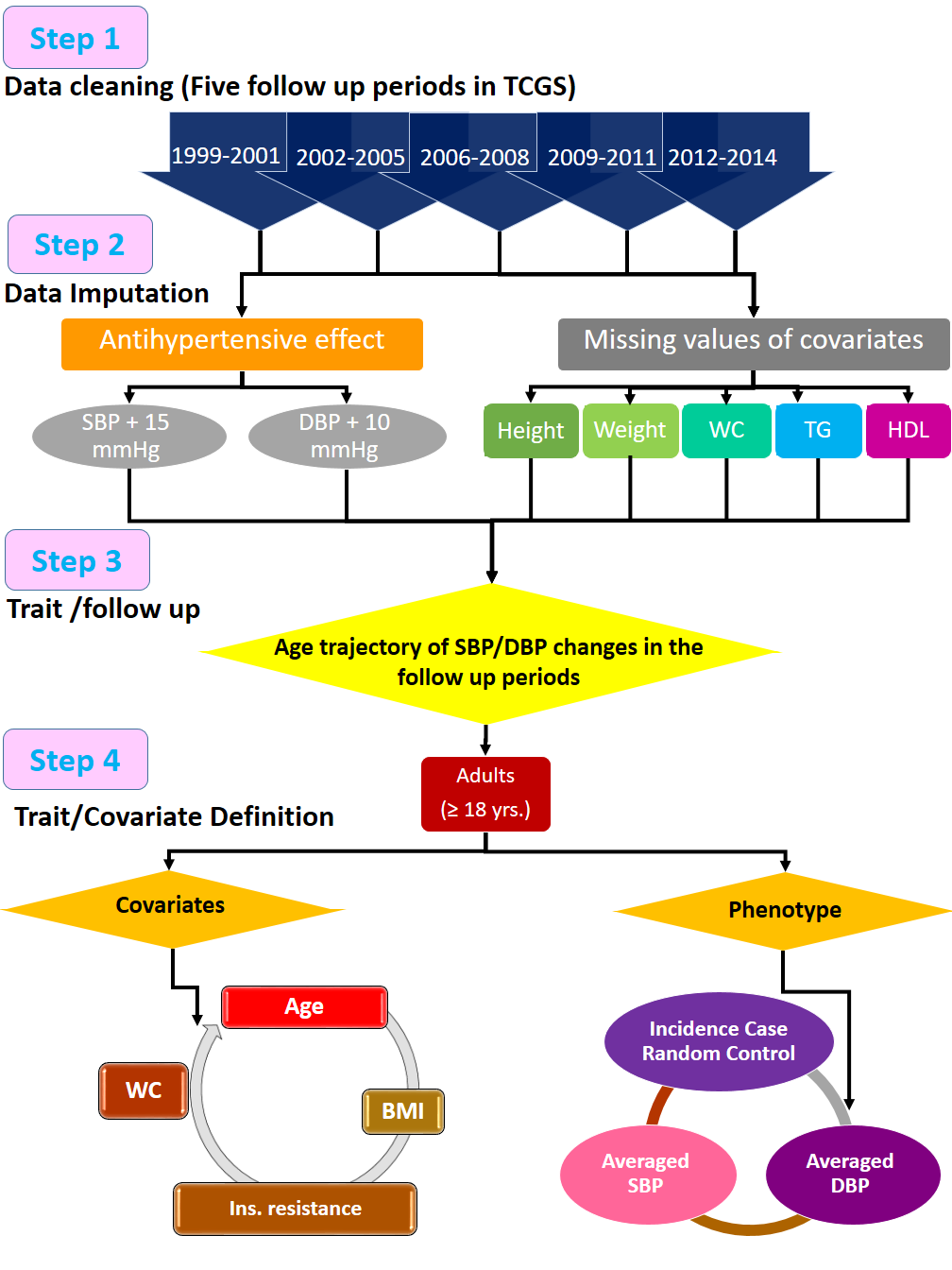


Supp.2. Distribution of SBP and DBP in five study phases by age.


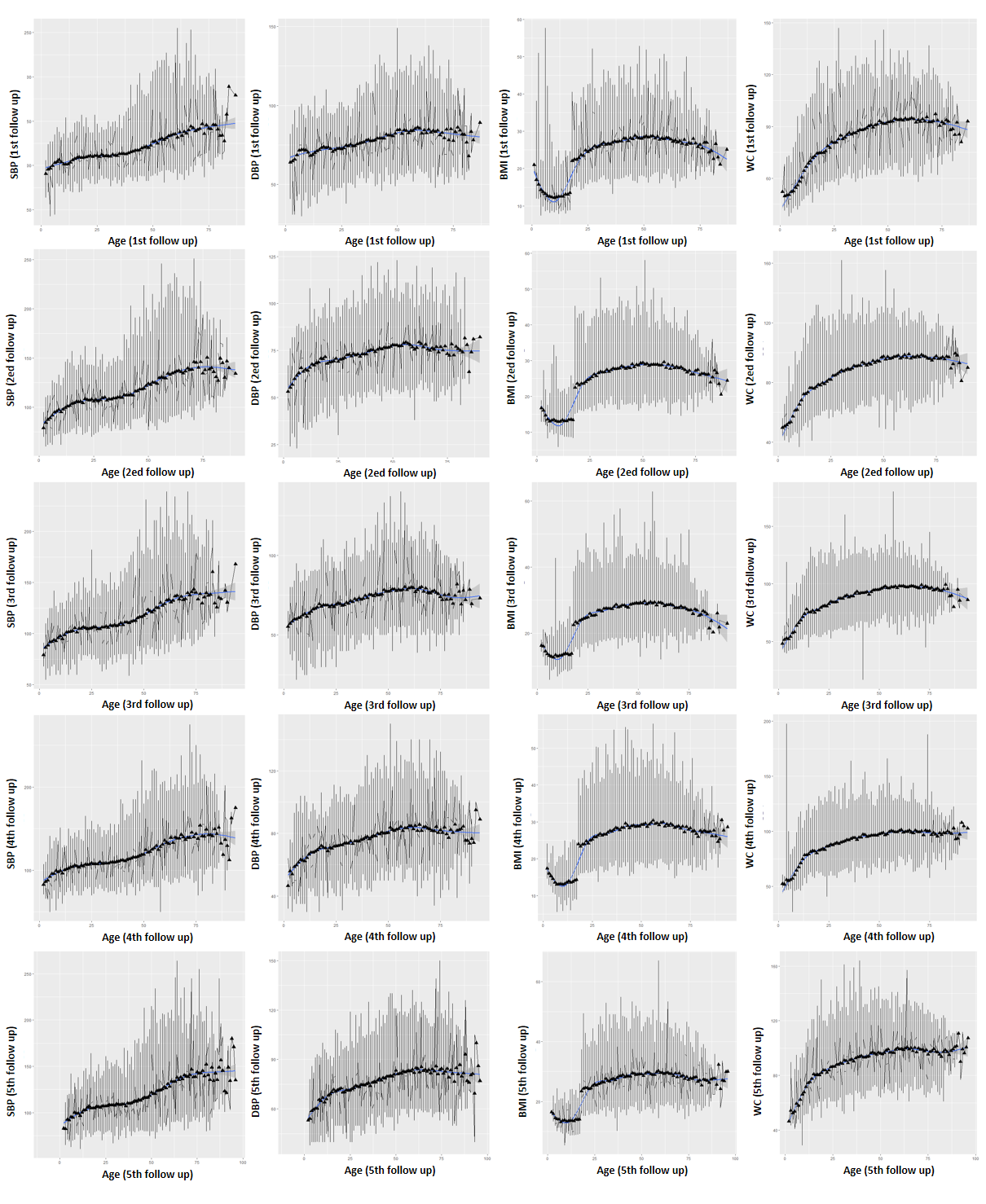


Supp.3. Sensitivity analysis of imputation values in five study phases (A-E)

| **A: First follow up** | | | | | |  |
| --- | --- | --- | --- | --- | --- | --- |
| **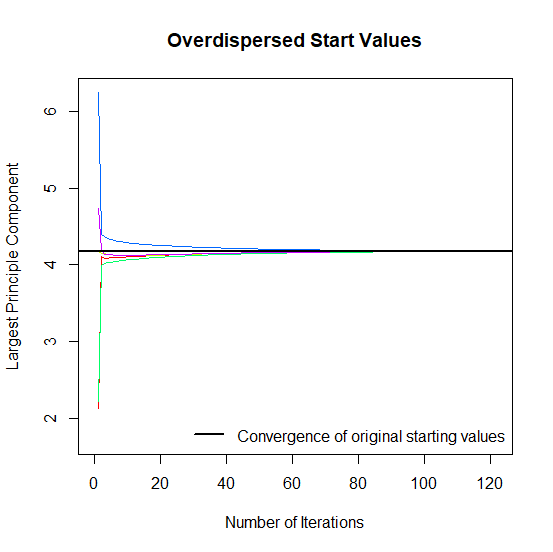** | | **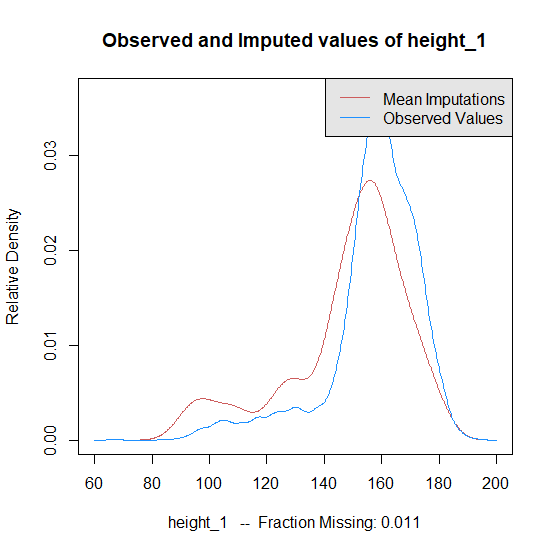** | | **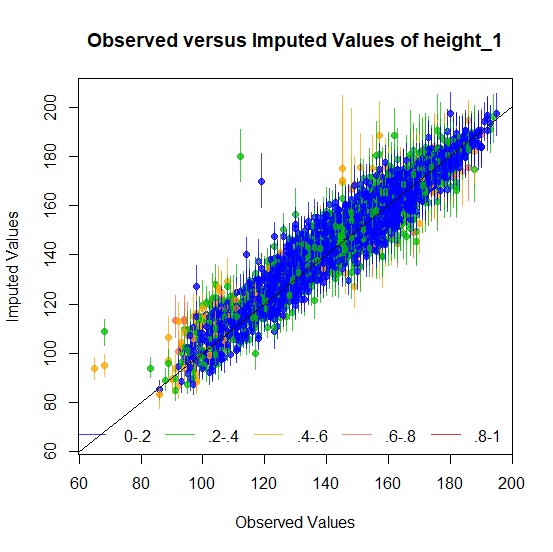** | |  |
| **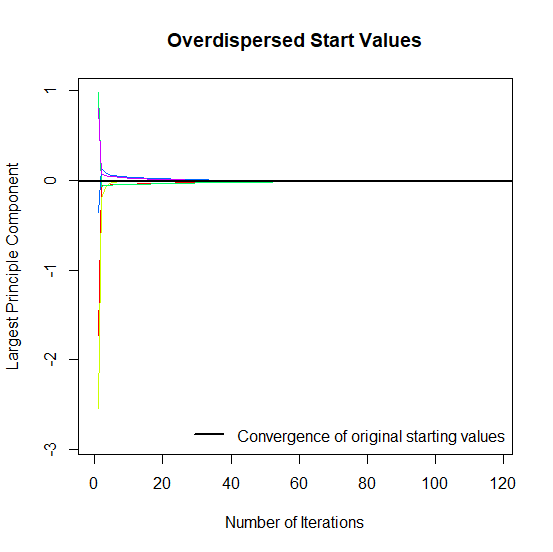** | | **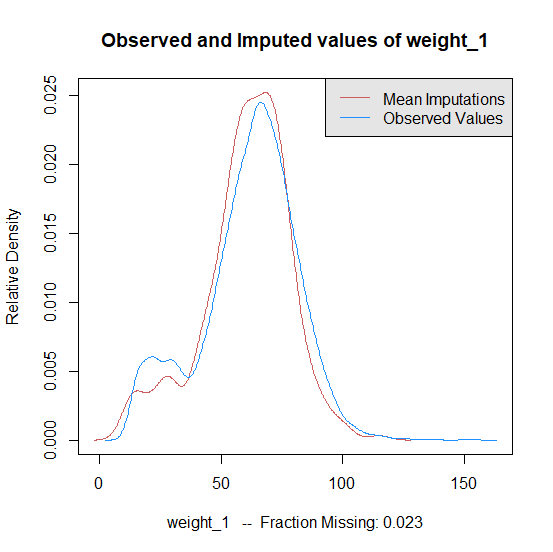** | | **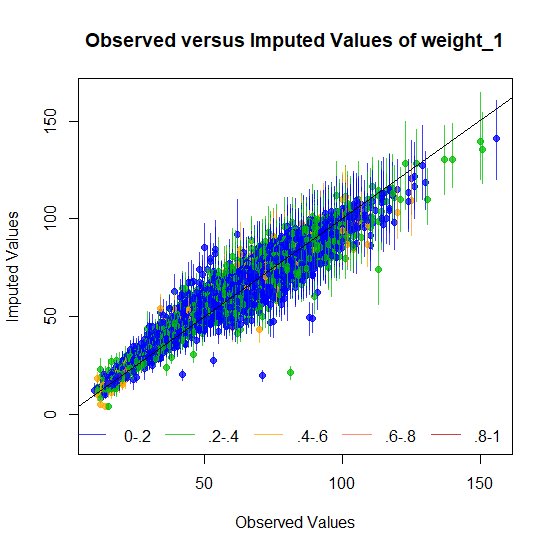** | |  |
| **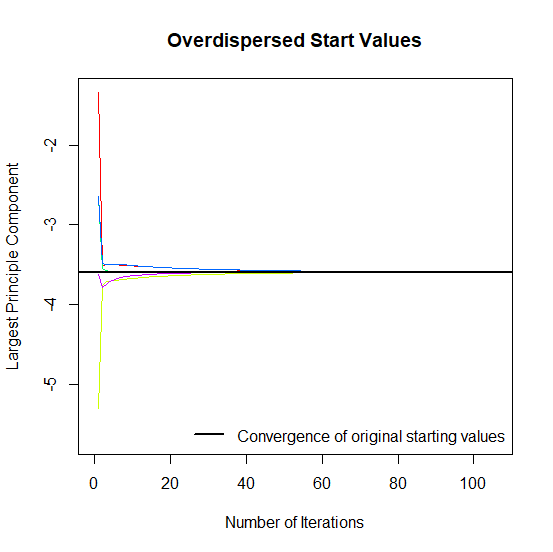** | | **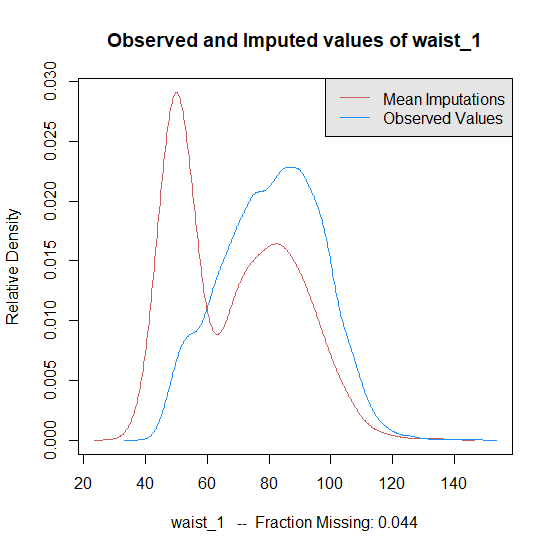** | | **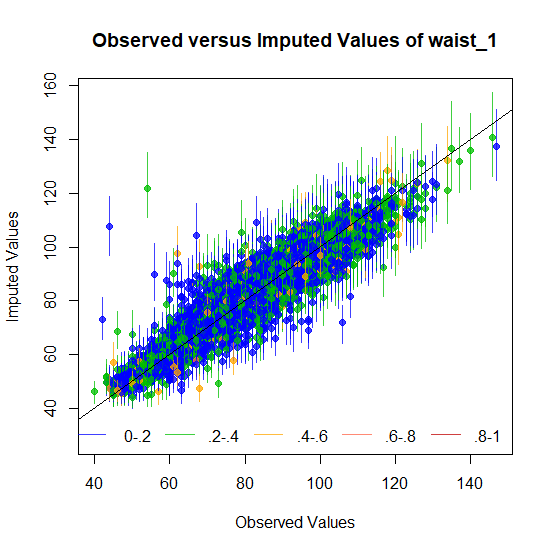** | |  |
| **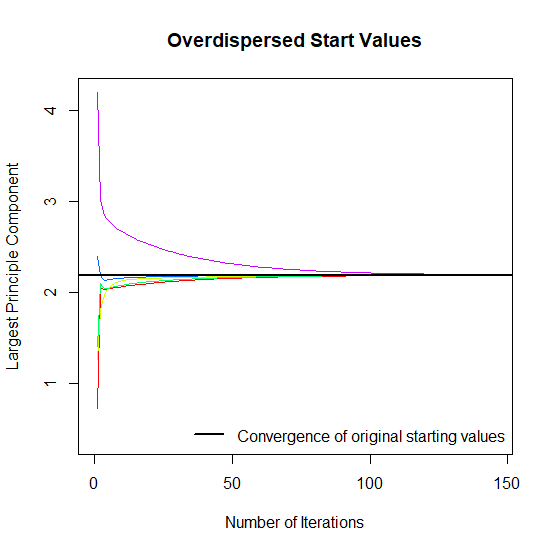** | | **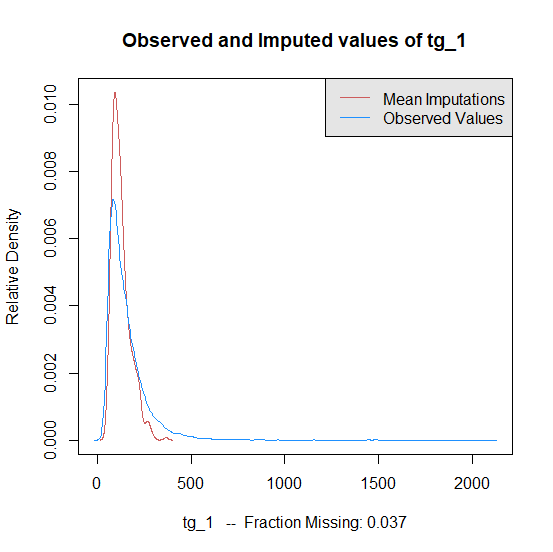** | | **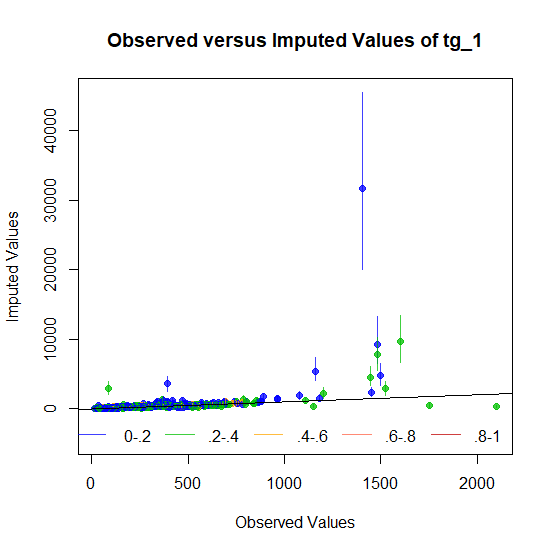** | |  |
| **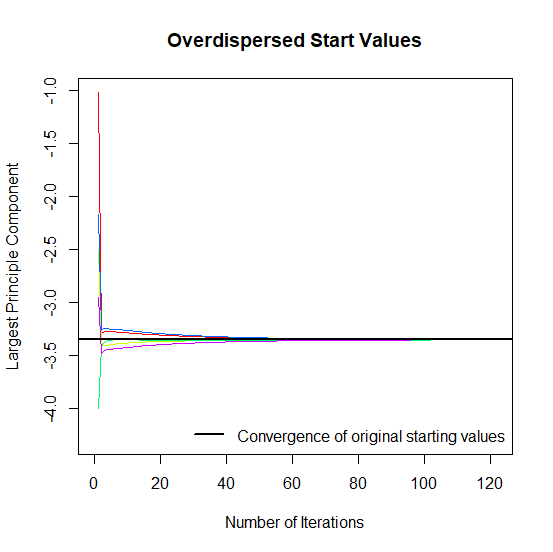** | | **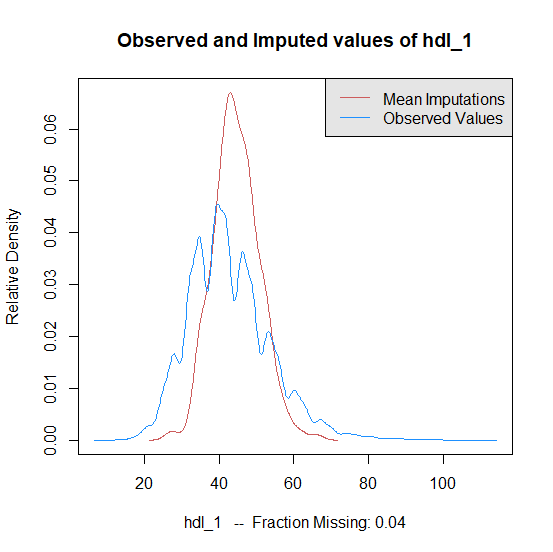** | | **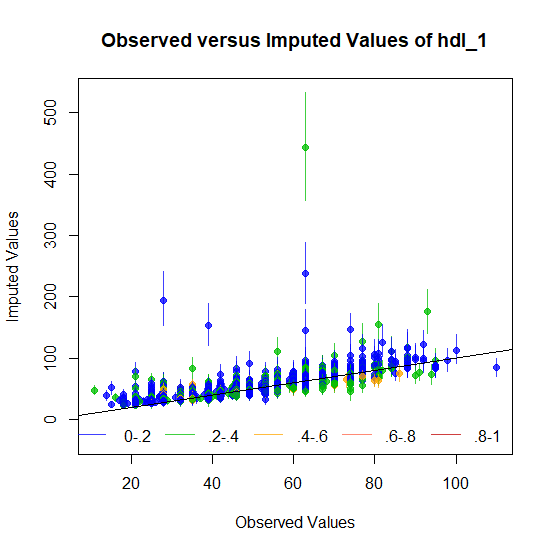** | |  |
| **B: Second follow up** | | | | | |  |
| **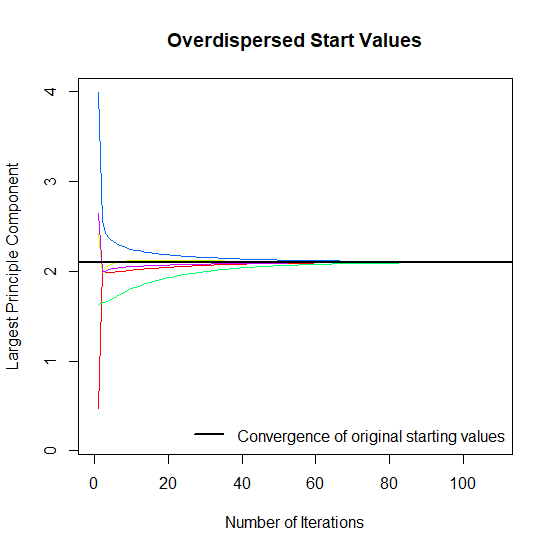** | | **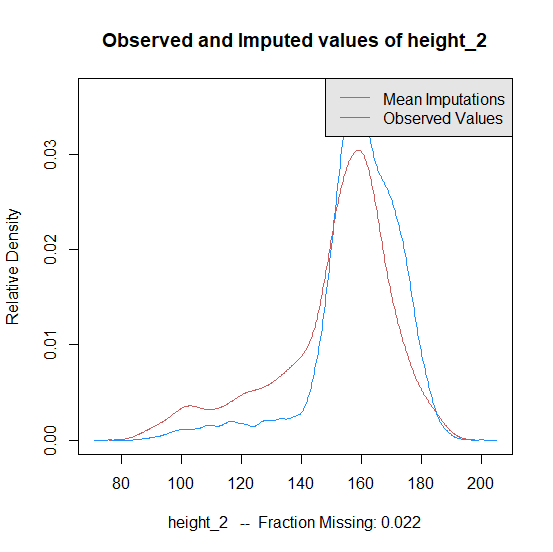** | | **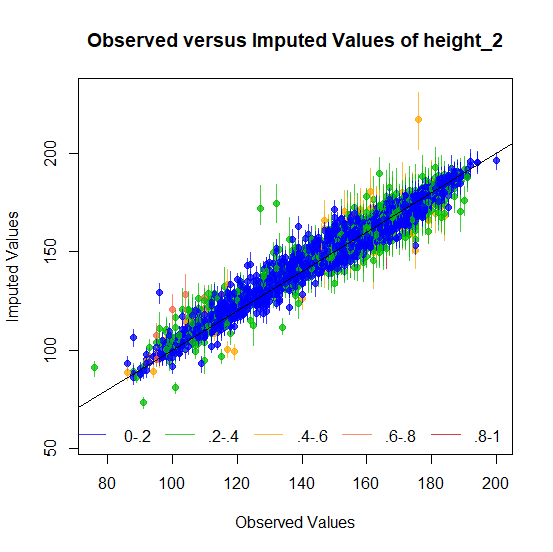** | |  |
| **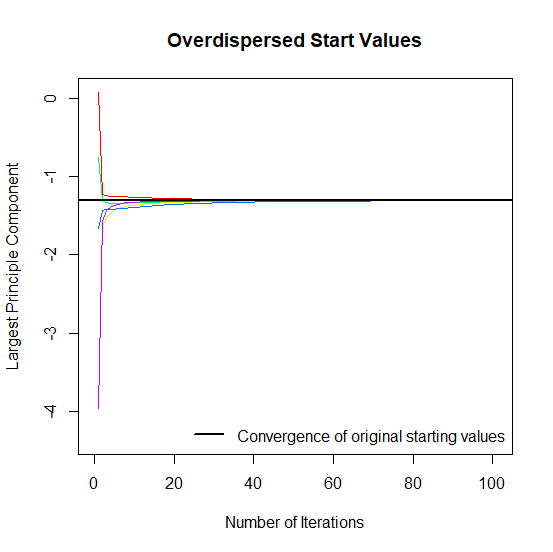** | | **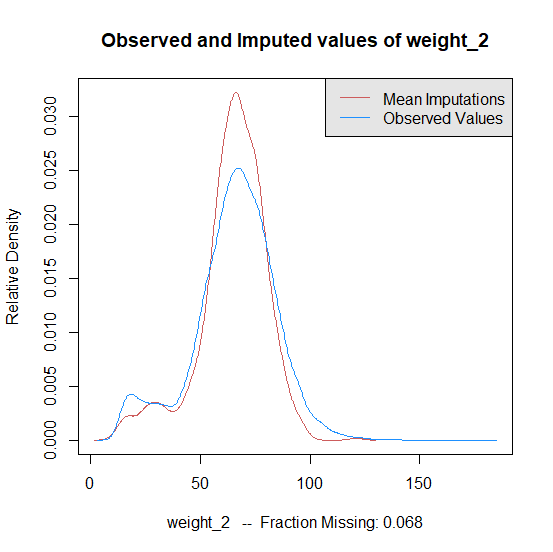** | | **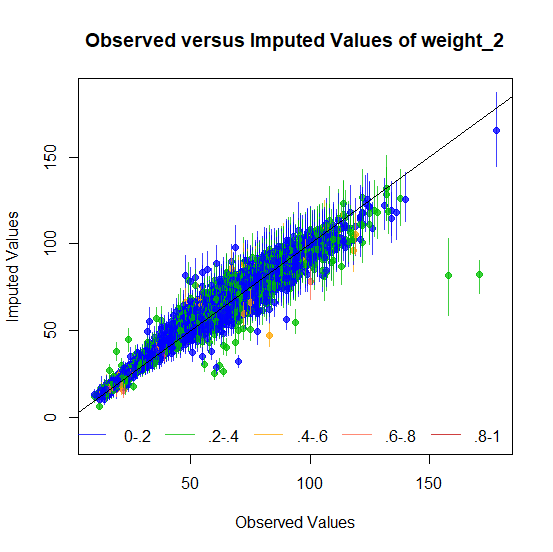** | |  |
| **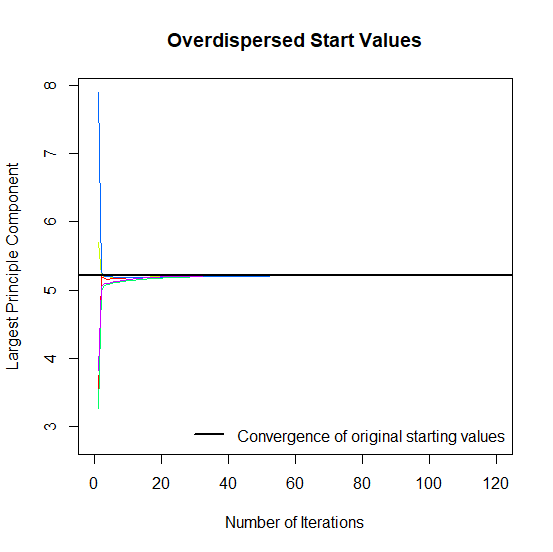** | | **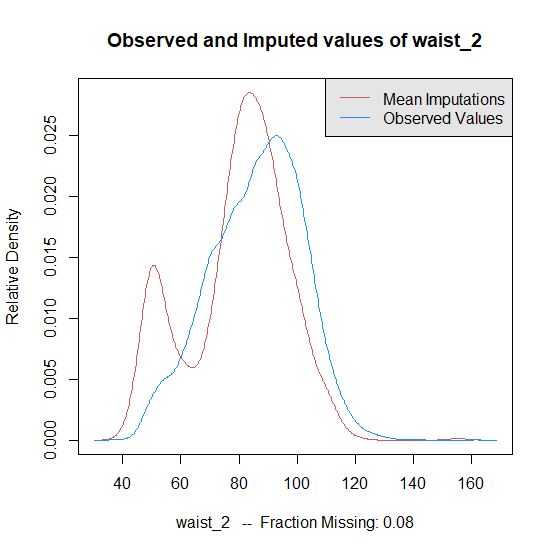** | | **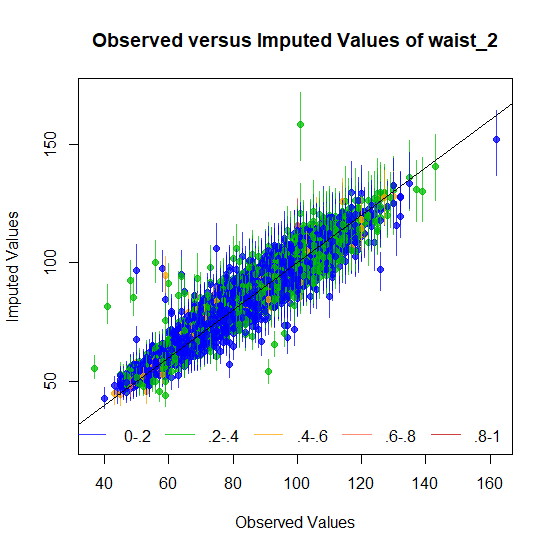** | |  |
| **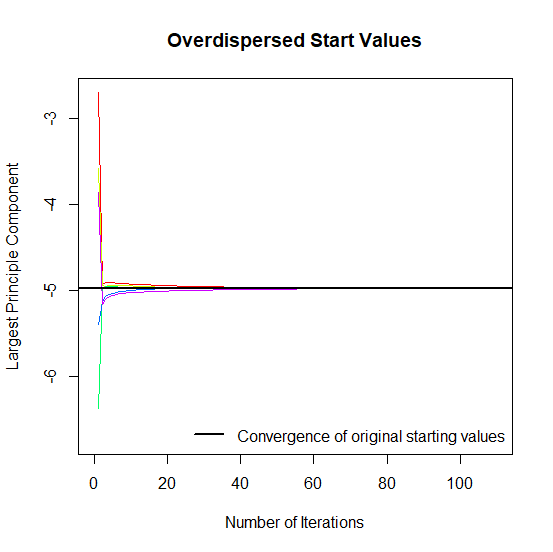** | | **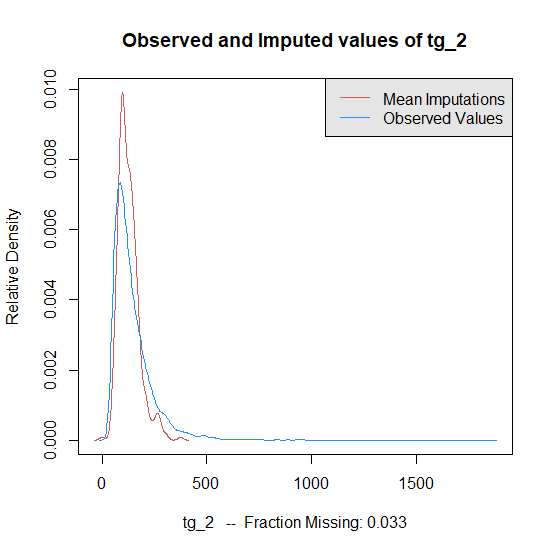** | | **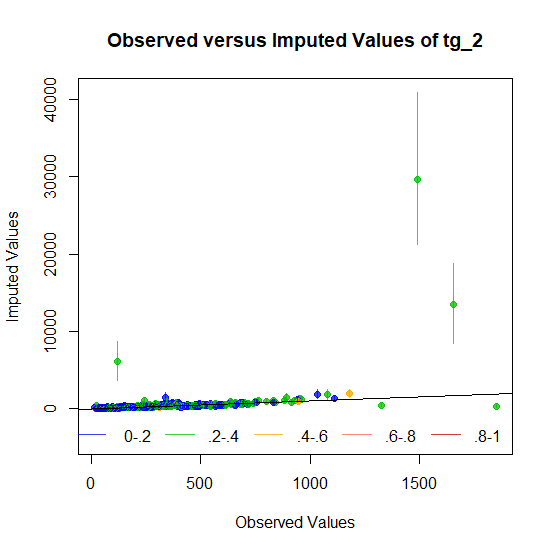** | |  |
| **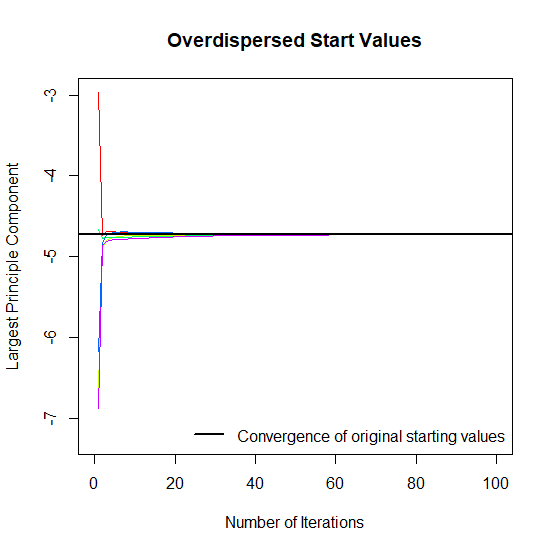** | | **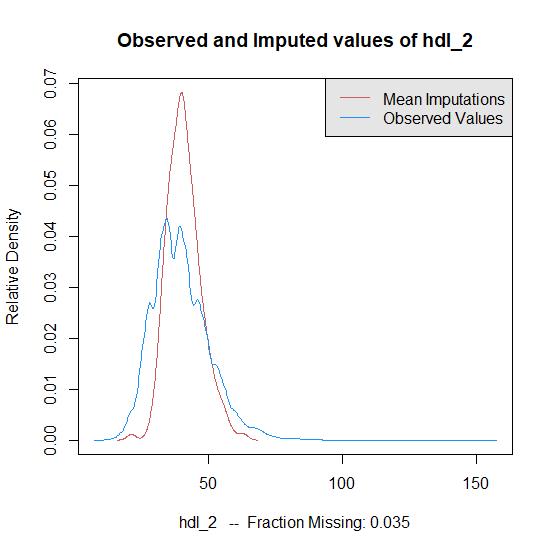** | | **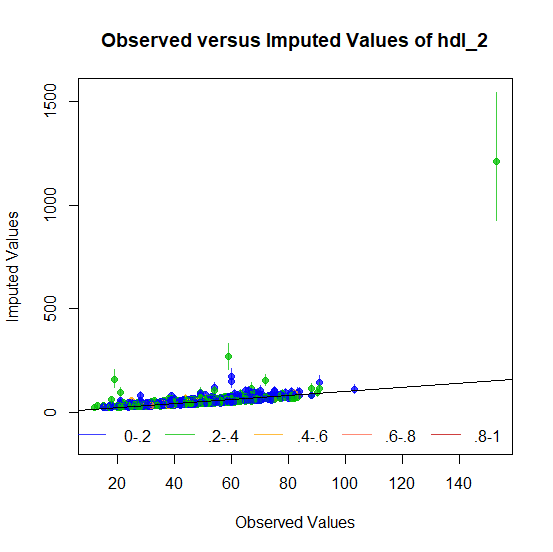** | |  |
| **C: Third follow up** | | | | | | |
| **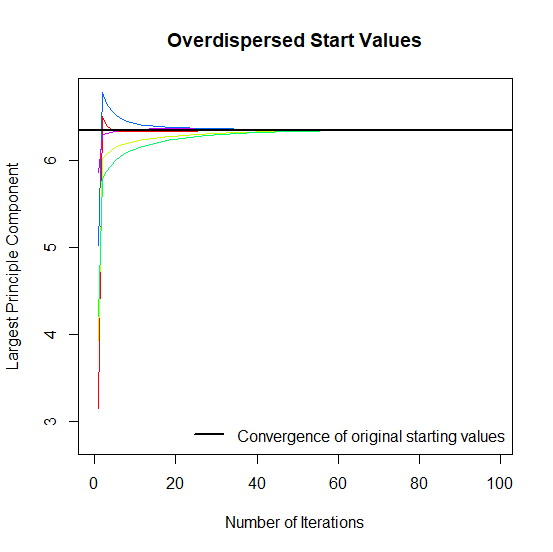** | | **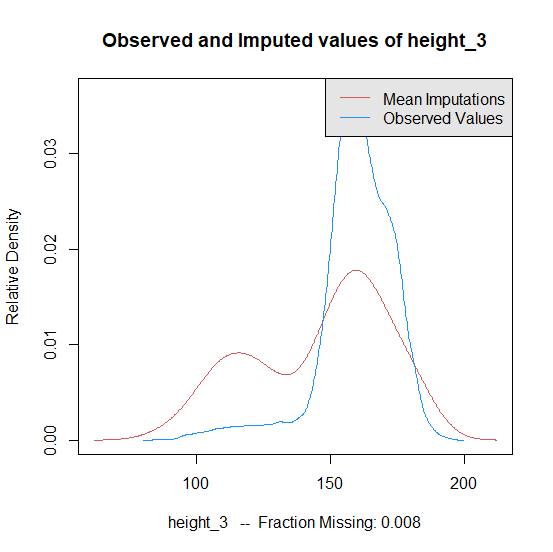** | | **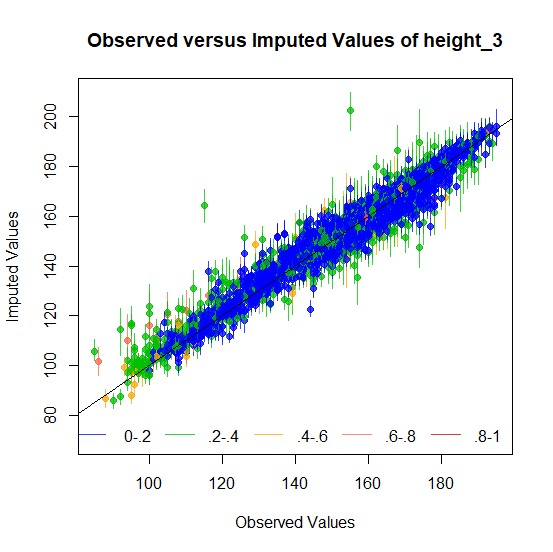** | | |
| **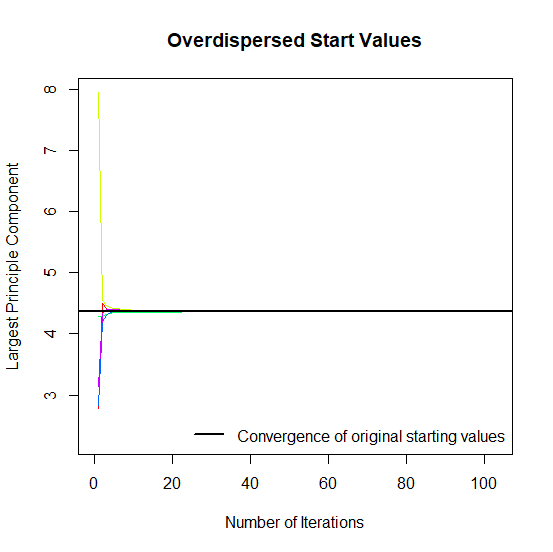** | | **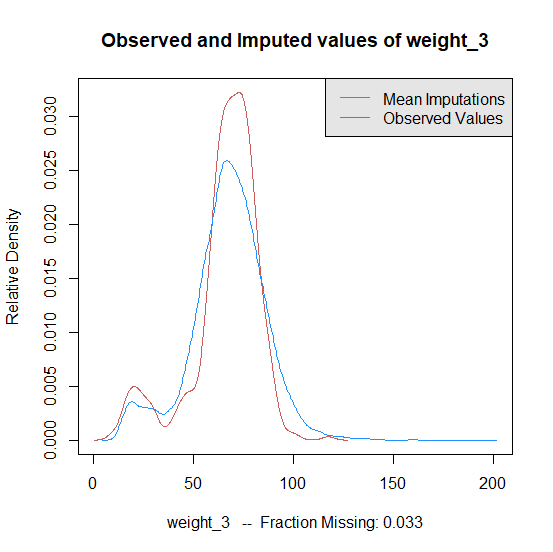** | | **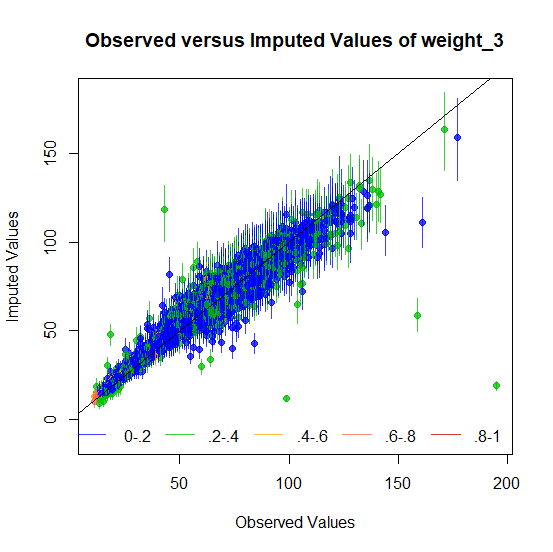** | | |
| **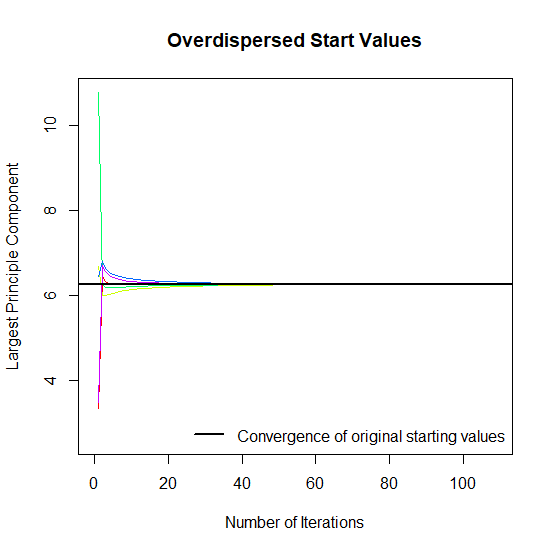** | | **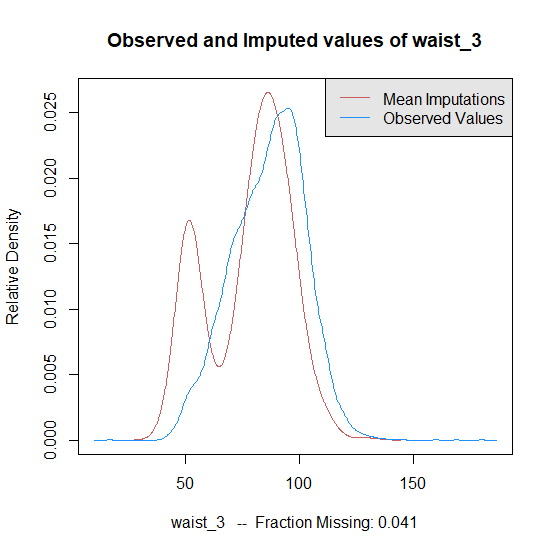** | | **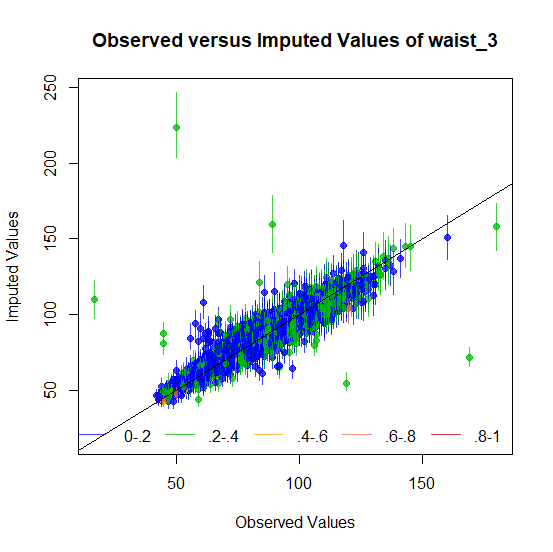** | | |
| **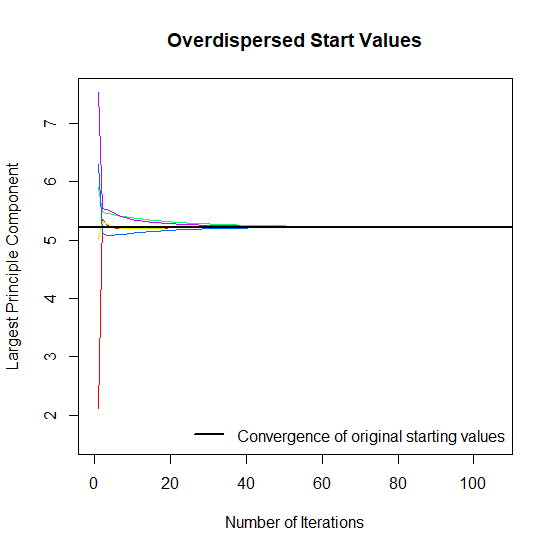** | | **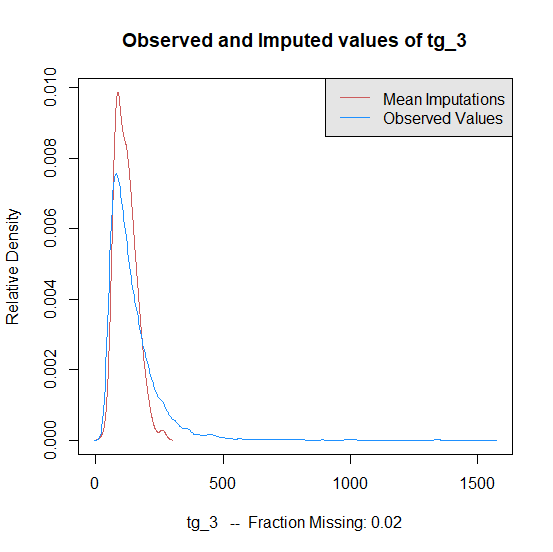** | | **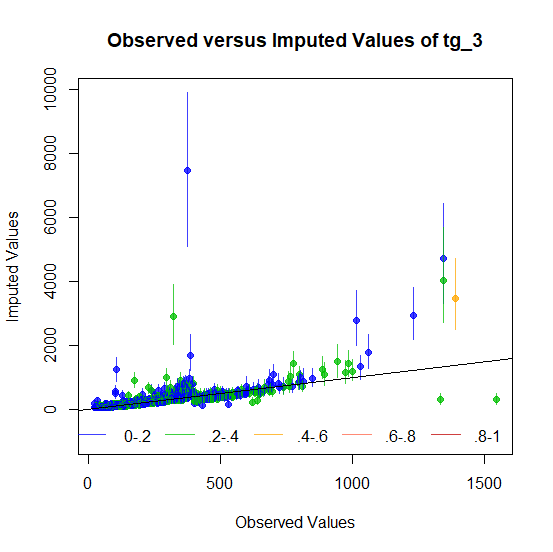** | | |
| **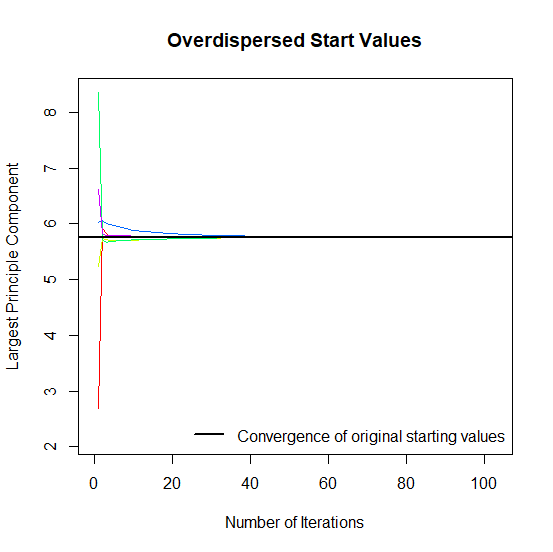** | | **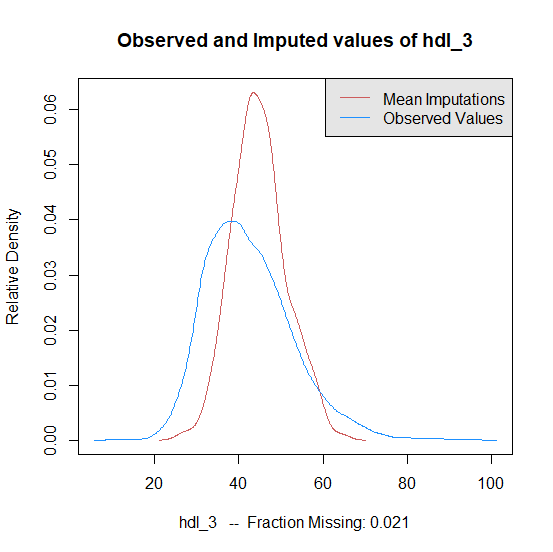** | | **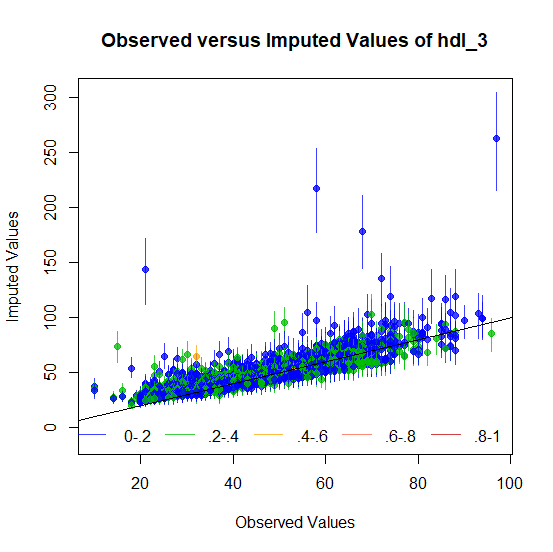** | | |

| **D: Fourth follow up** | | |  |
| --- | --- | --- | --- |
| **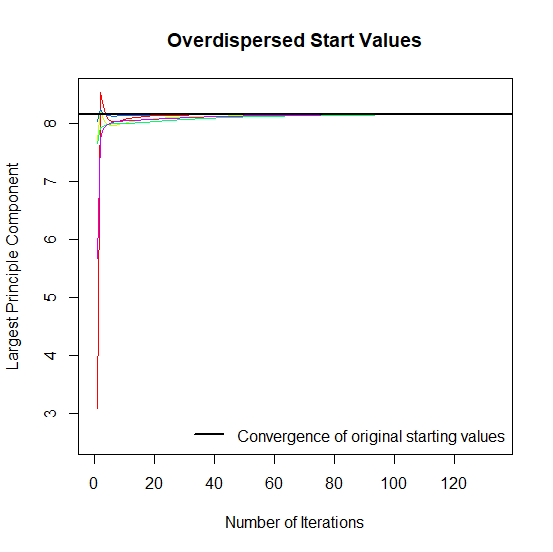** | **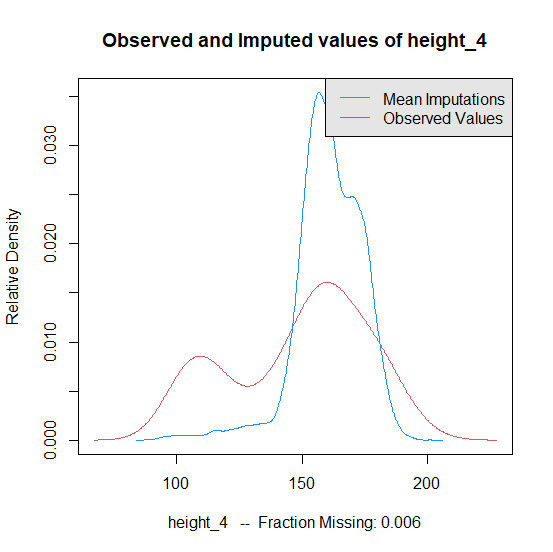** | **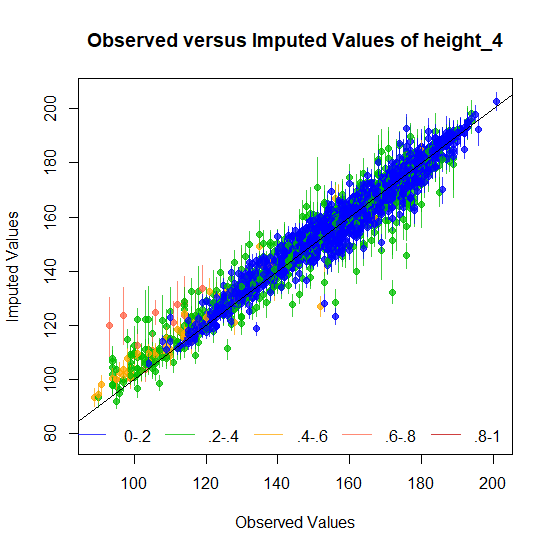** |  |
| **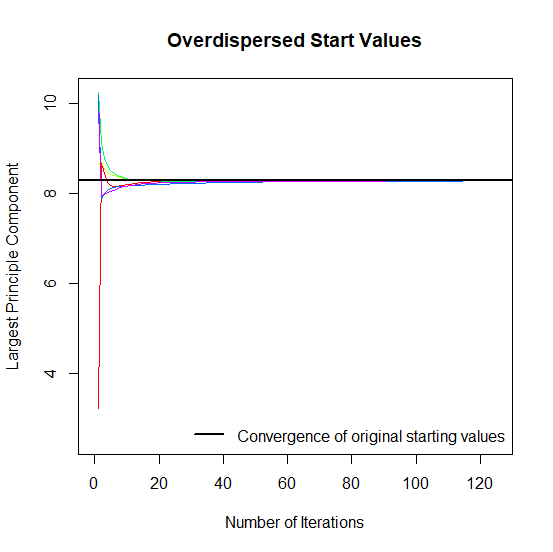** | **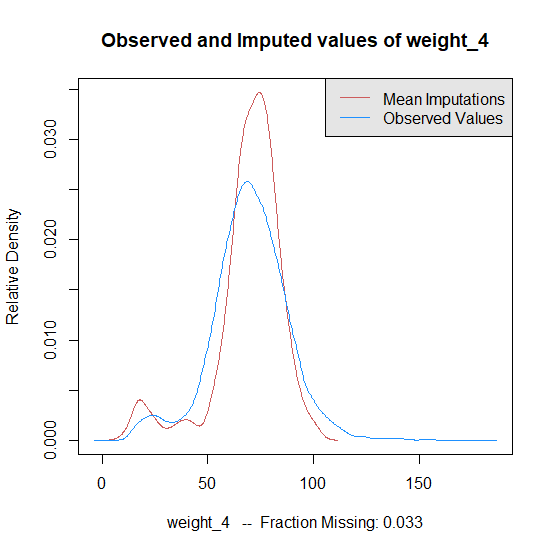** | **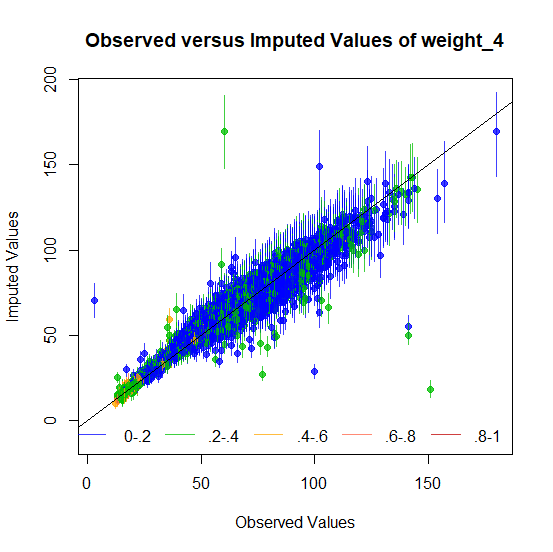** |  |
| **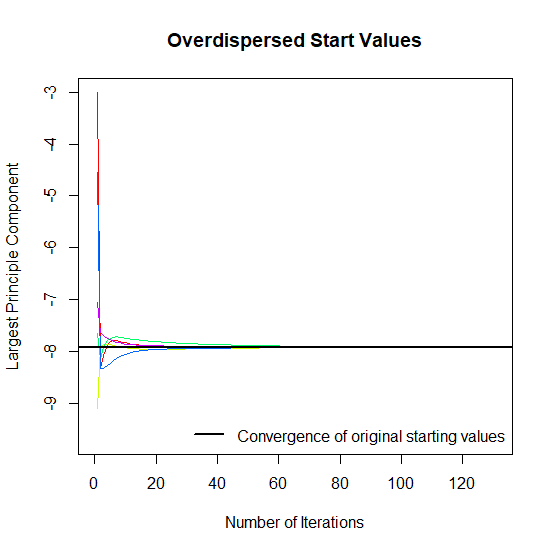** | **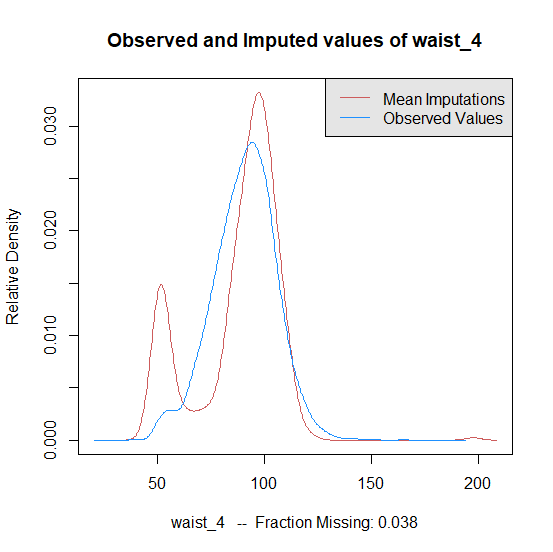** | **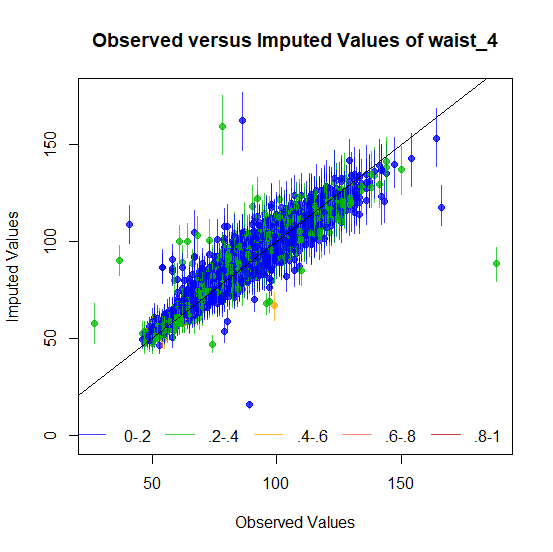** |  |
| **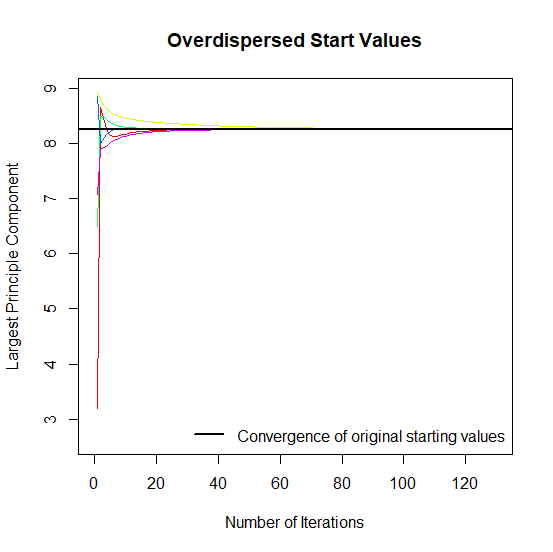** | **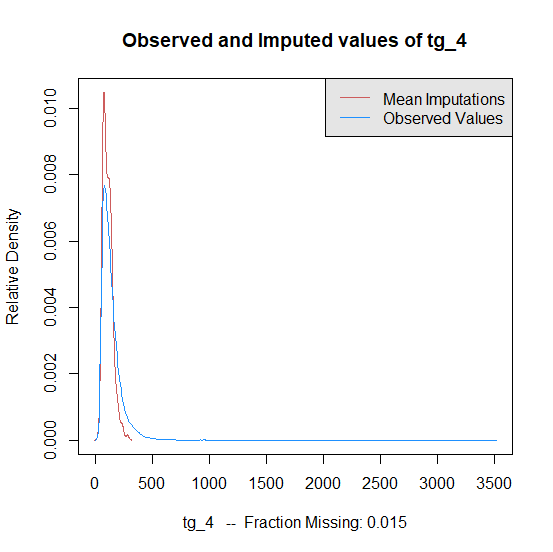** | **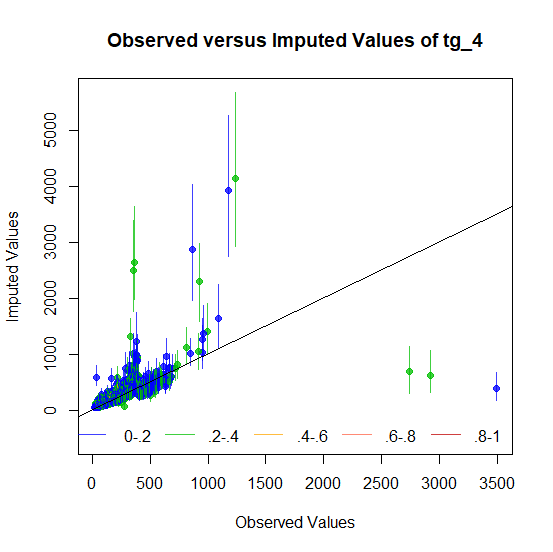** |  |
| **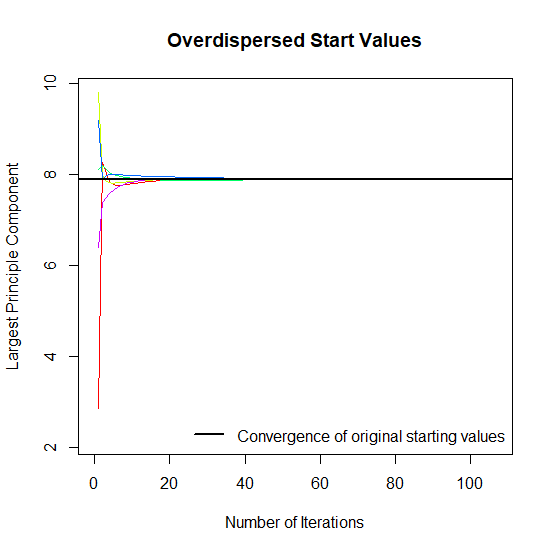** | **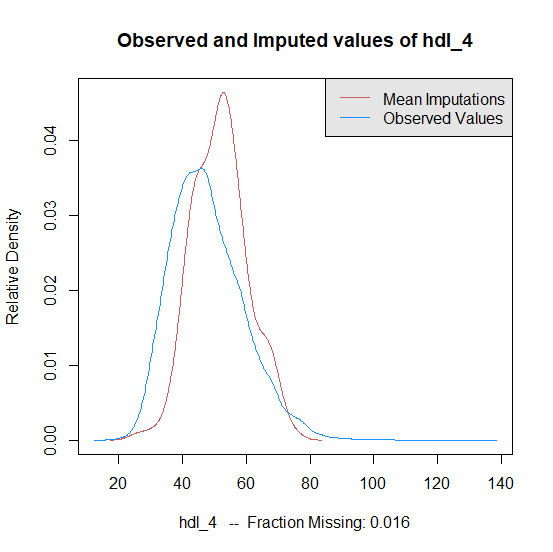** | **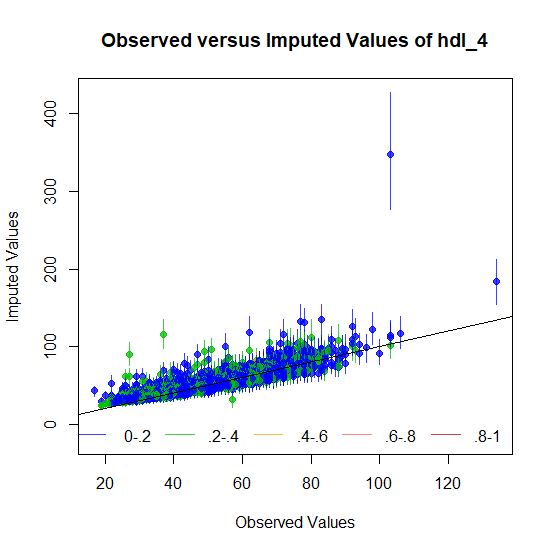** |  |
| **E: Fifth follow up** | | | |
| **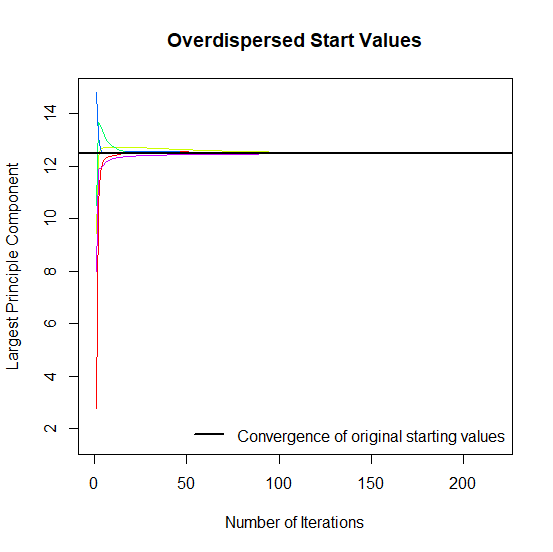** | **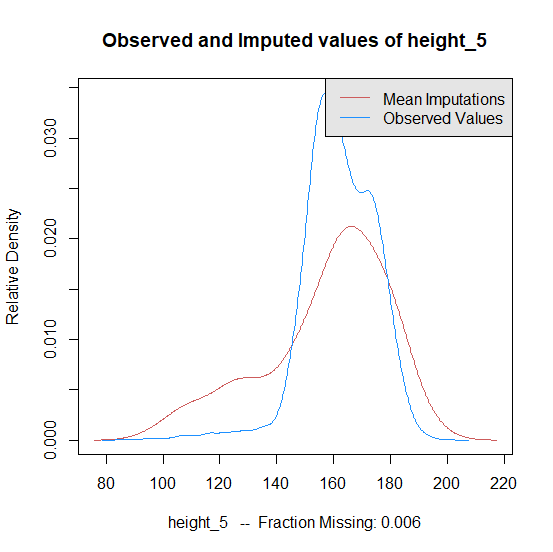** | **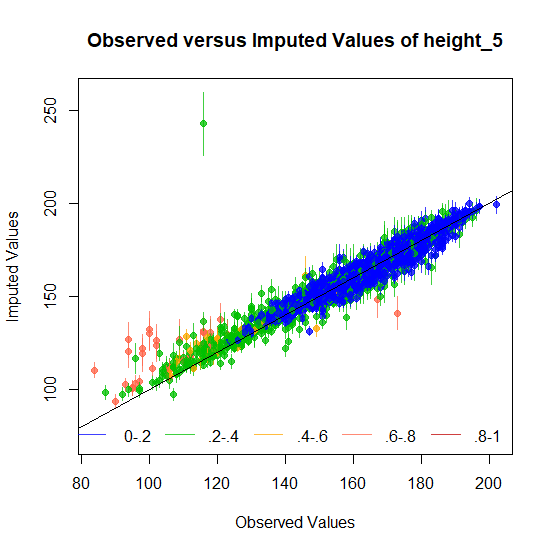** | |
| **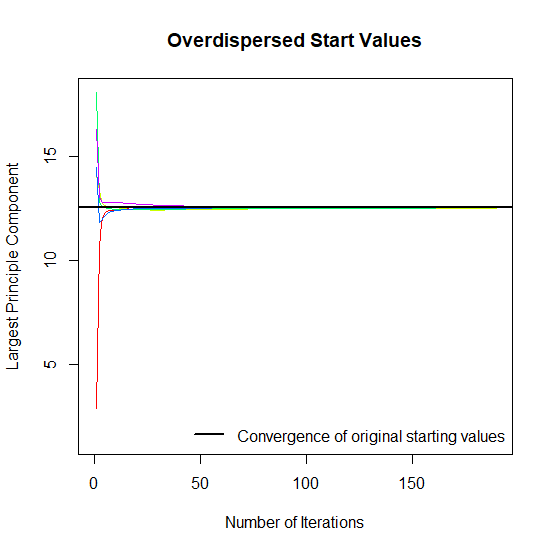** | **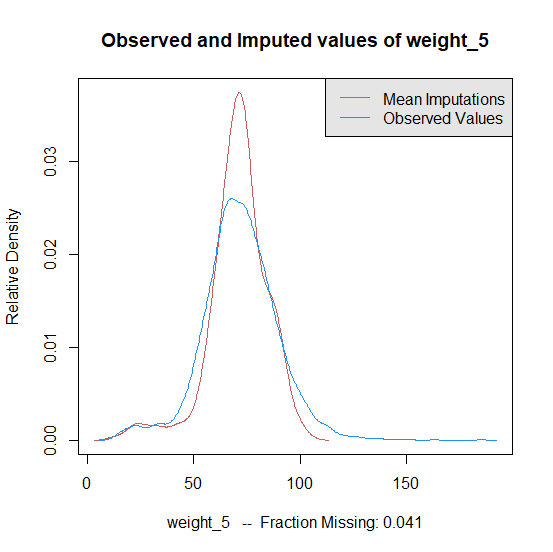** | **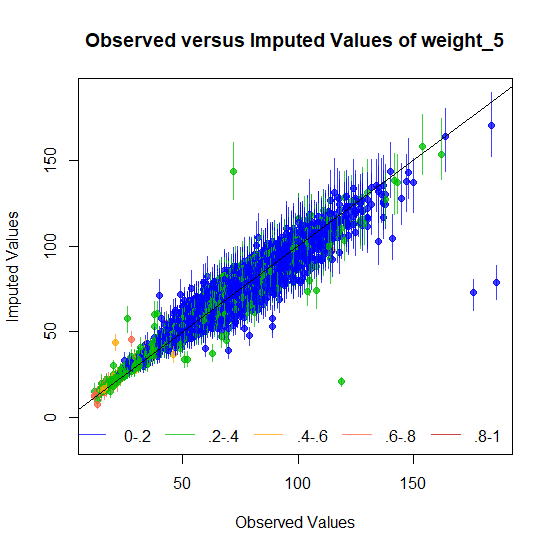** | |
| **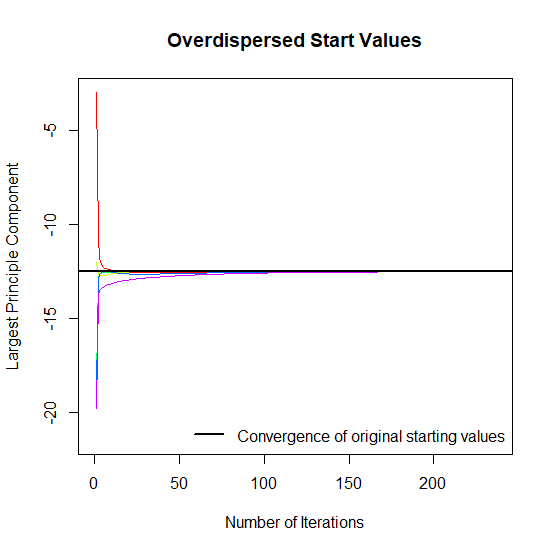** | **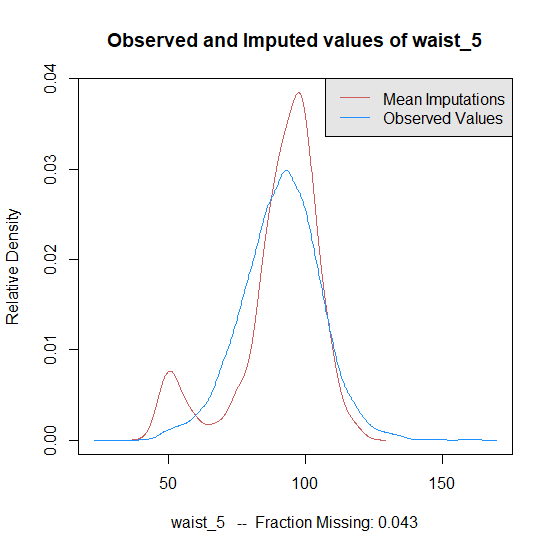** | **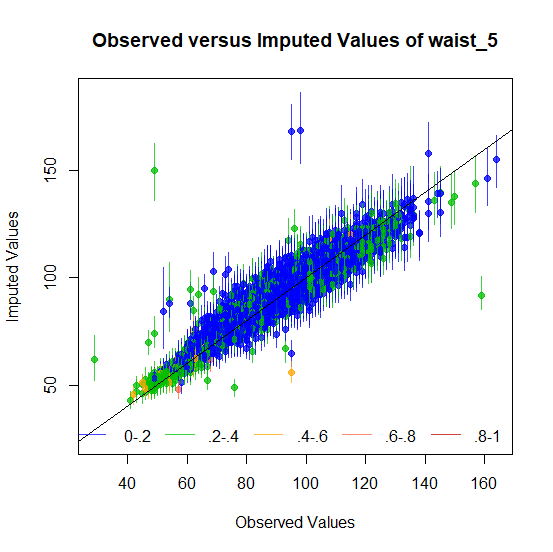** | |
| **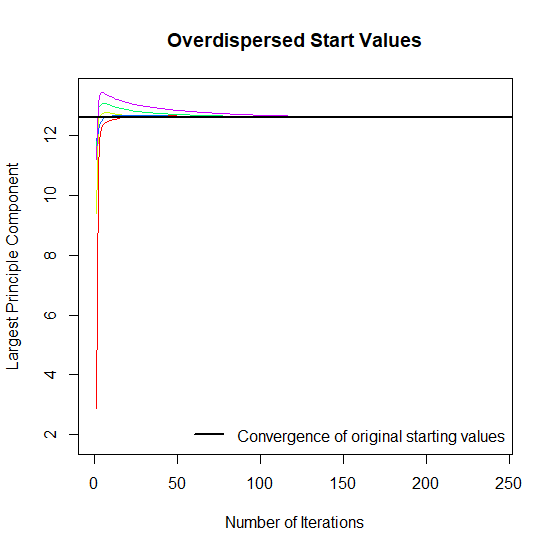** | **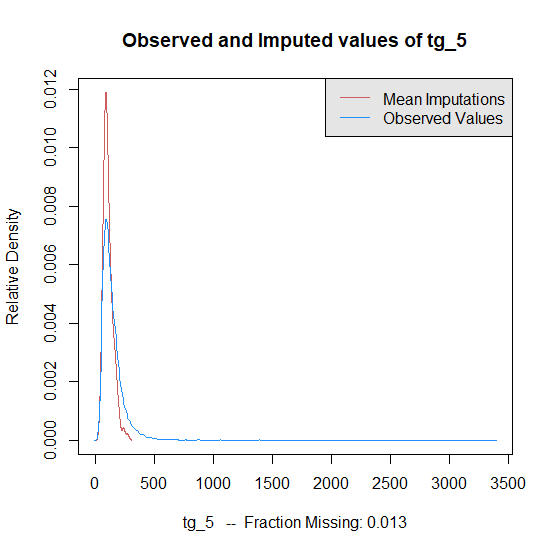** | **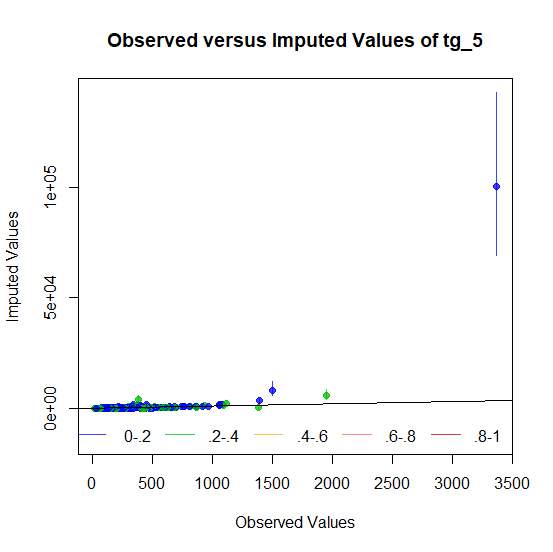** | |
| **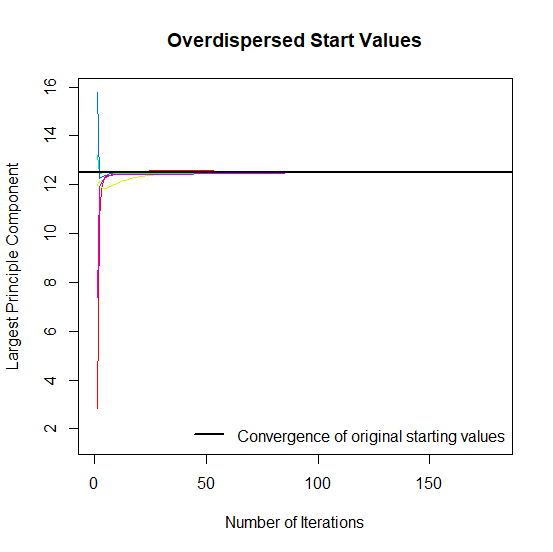** | **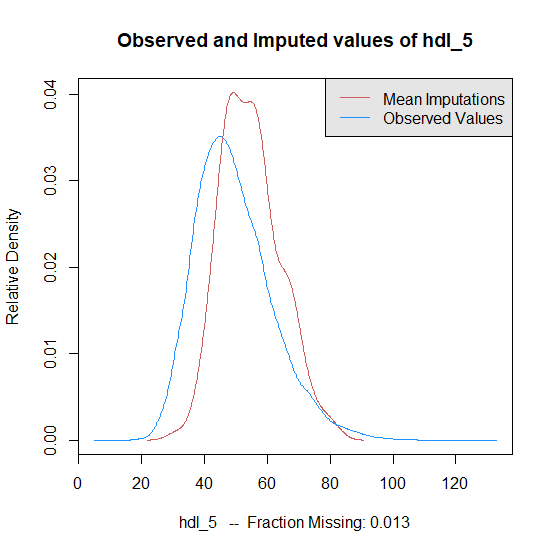** | **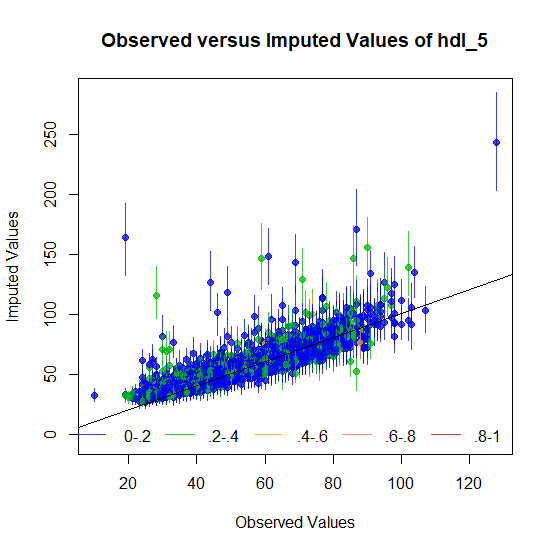** | |

Supp.4. QC pipeline for quantitative and binary traits

| 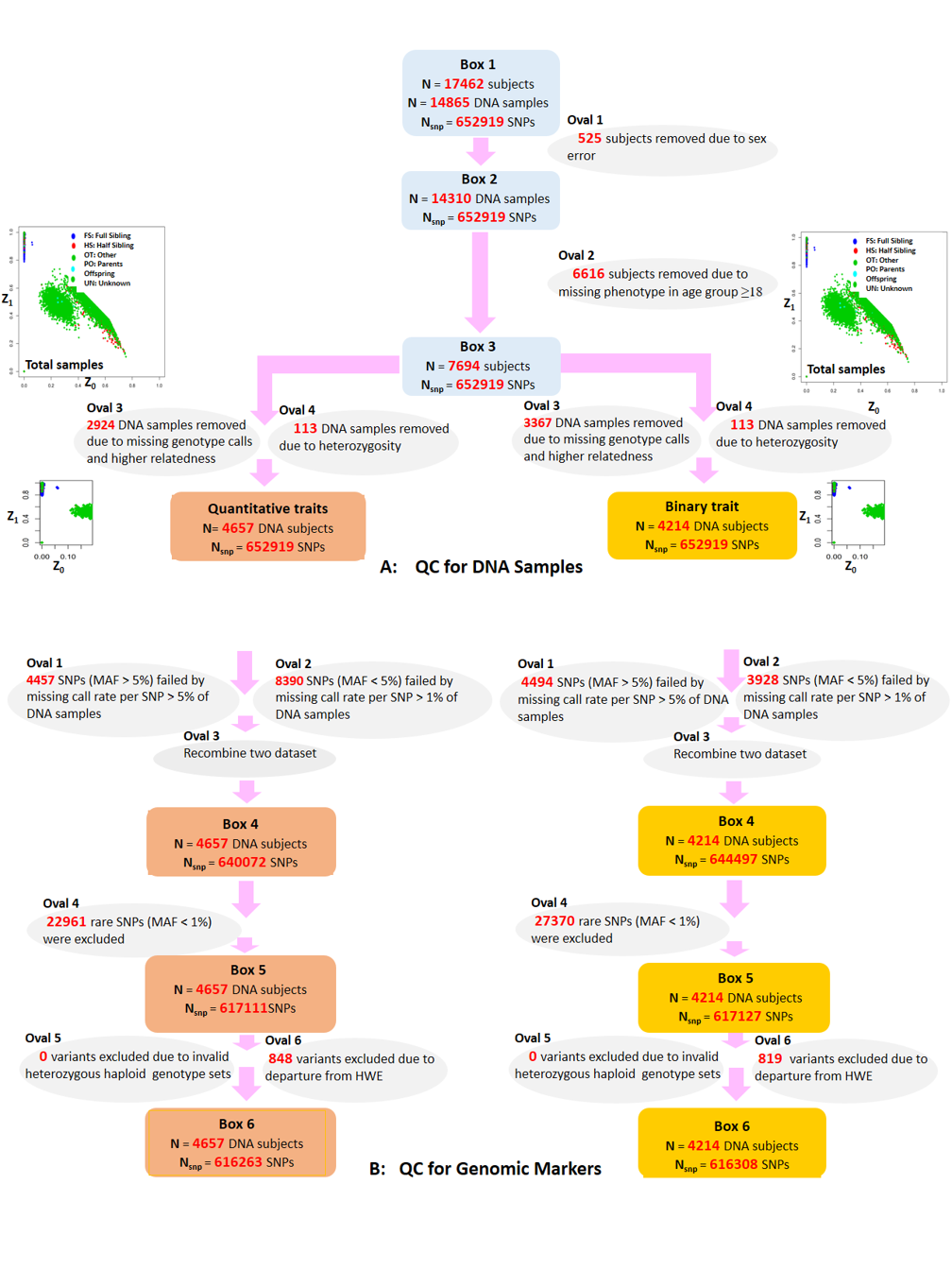 |
| --- |
|  |

Supp.5. Trait-covariate correlation.


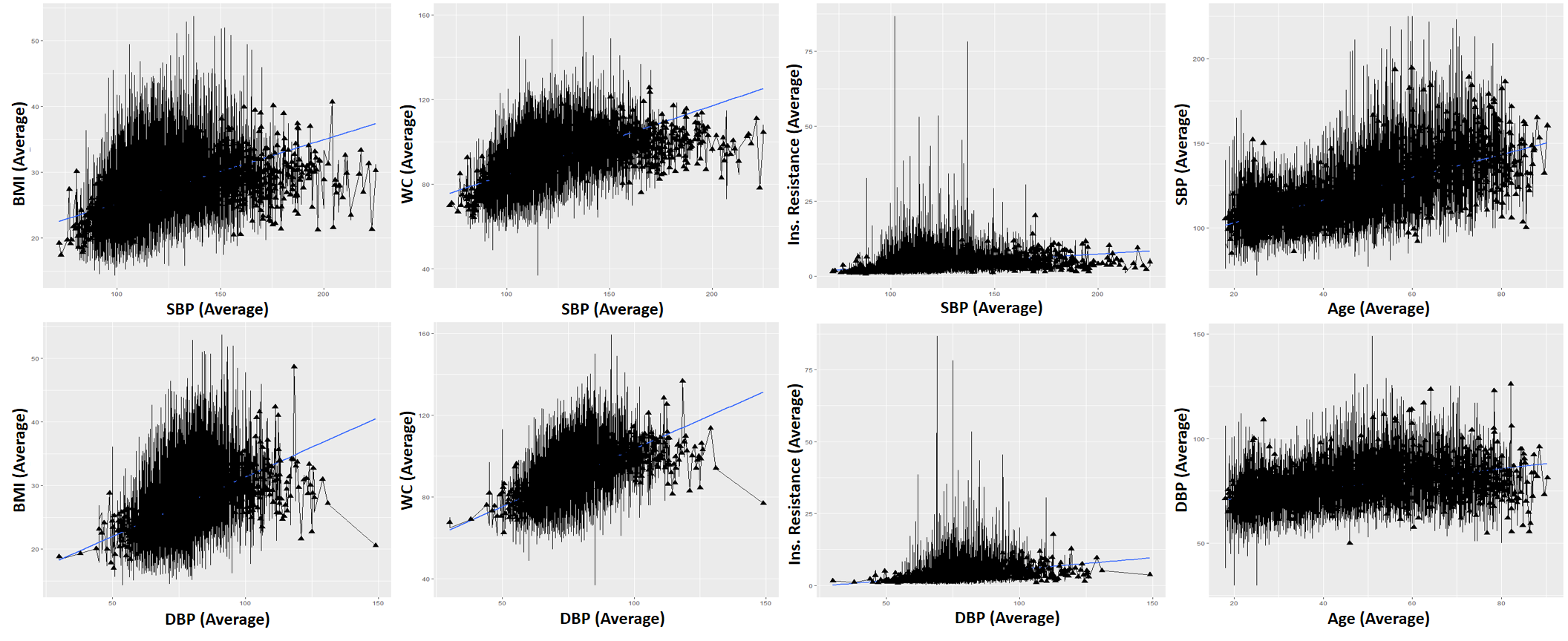


Supp.6. Covariates correlation.


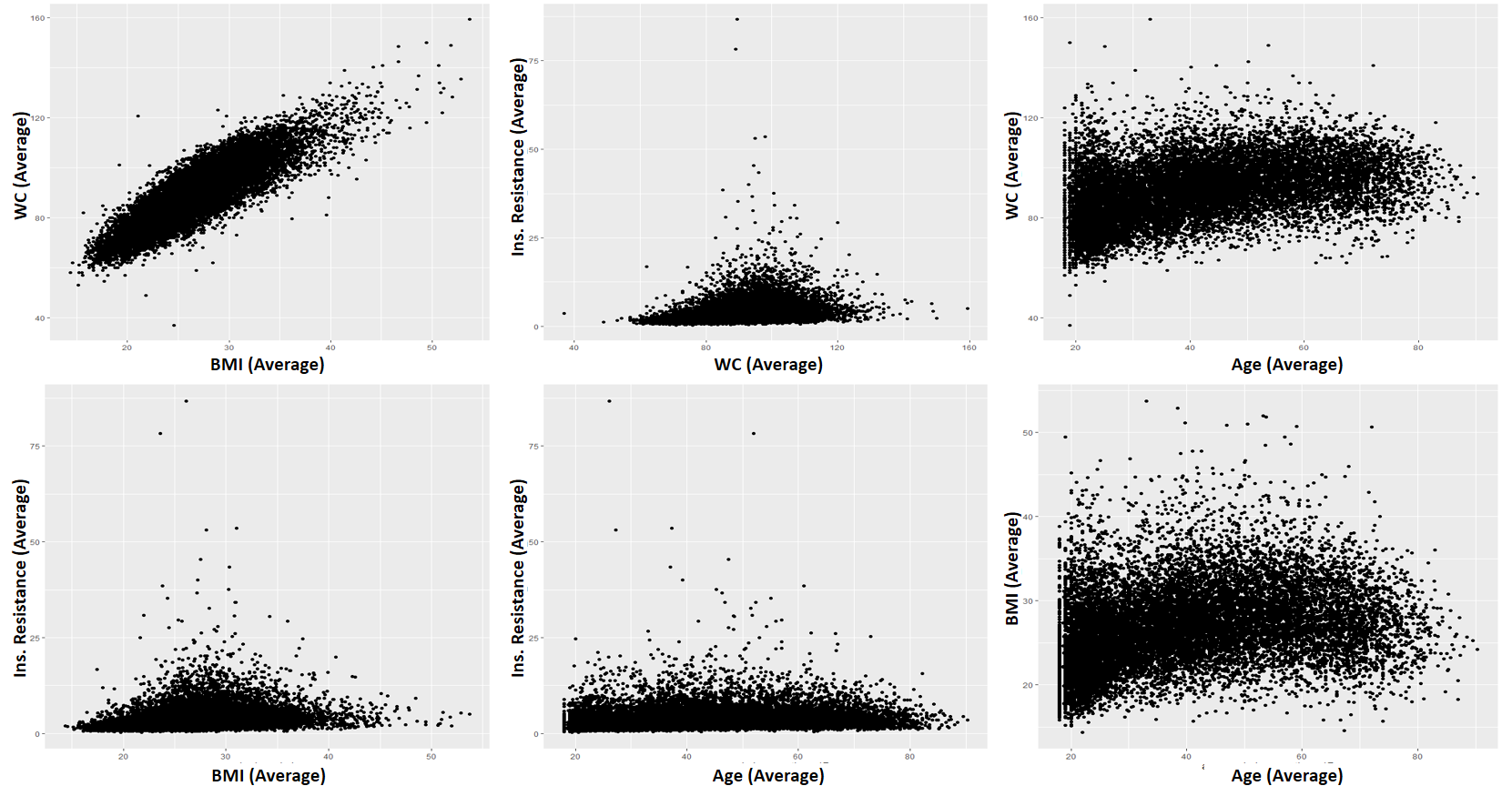


Supp.7. Previously reported associations between BP traits or cardiovascular diseases and detected loci in GWAS.


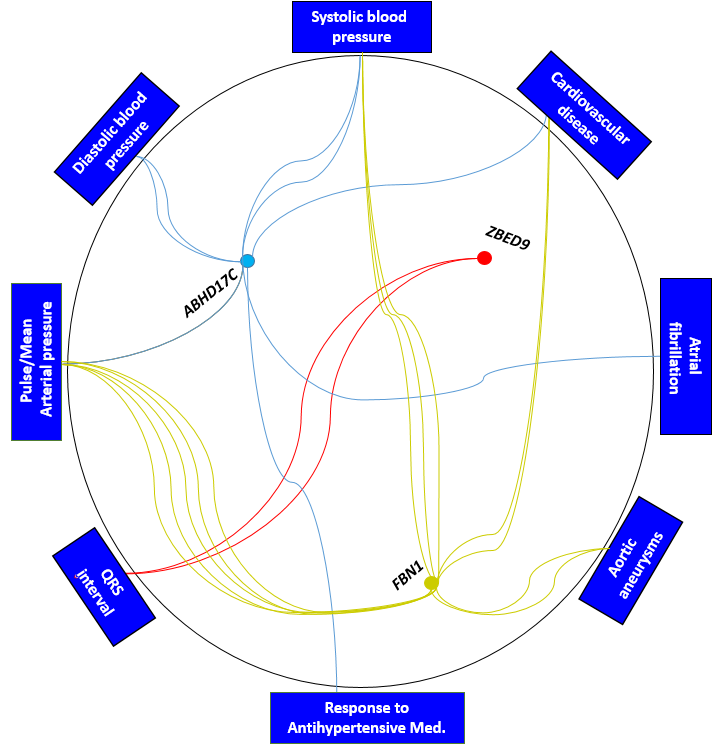

Supplement: Supplementary file 1 — Supplementary Information 1. [file 41598_2021_90925_MOESM1_ESM.docx]
